# The Structural Biology of Septins and Their Filaments: An Update

**DOI:** 10.3389/fcell.2021.765085

**Published:** 2021-11-19

**Authors:** Italo A. Cavini, Diego A. Leonardo, Higor V. D. Rosa, Danielle K. S. V. Castro, Humberto D’Muniz Pereira, Napoleão F. Valadares, Ana P. U. Araujo, Richard C. Garratt

**Affiliations:** ^1^ São Carlos Institute of Physics, University of São Paulo, São Carlos, Brazil; ^2^ São Carlos Institute of Chemistry, University of São Paulo, São Carlos, Brazil; ^3^ Department of Cellular Biology, University of Brasília, Brasília, Brazil

**Keywords:** septin, structural biology, cytoskeletal protein, protein filament, hetero-oligomeric complex, GTP-binding domain, coiled coil

## Abstract

In order to fully understand any complex biochemical system from a mechanistic point of view, it is necessary to have access to the three-dimensional structures of the molecular components involved. Septins and their oligomers, filaments and higher-order complexes are no exception. Indeed, the spontaneous recruitment of different septin monomers to specific positions along a filament represents a fascinating example of subtle molecular recognition. Over the last few years, the amount of structural information available about these important cytoskeletal proteins has increased dramatically. This has allowed for a more detailed description of their individual domains and the different interfaces formed between them, which are the basis for stabilizing higher-order structures such as hexamers, octamers and fully formed filaments. The flexibility of these structures and the plasticity of the individual interfaces have also begun to be understood. Furthermore, recently, light has been shed on how filaments may bundle into higher-order structures by the formation of antiparallel coiled coils involving the C-terminal domains. Nevertheless, even with these advances, there is still some way to go before we fully understand how the structure and dynamics of septin assemblies are related to their physiological roles, including their interactions with biological membranes and other cytoskeletal components. In this review, we aim to bring together the various strands of structural evidence currently available into a more coherent picture. Although it would be an exaggeration to say that this is complete, recent progress seems to suggest that headway is being made in that direction.

## 1 Introduction

Since the first identification of septins in *Saccharomyces cerevisiae*, more than 50 years ago by Hartwell (Hartwell, 1971), studies regarding the role they play in the cell have contributed to highlighting their fascinating properties. The initial studies in yeast cells showed that septins appeared to be membrane associated and formed a collar at the budding neck, important for recruiting proteins for cell division (Byers and Goetsch, 1976; Longtine et al., 2000; Finnigan et al., 2016; Tamborrini et al., 2018). Nowadays, it is known that in animal cells, septins may be found at a variety of locations, depending on the type and stage of cell development and may also act to restrict the diffusion of membrane components and rigidify the cell cortex at specific sites (Barral et al., 2000; Spiliotis and Gladfelter, 2012; Palander et al., 2017; Spiliotis, 2018; Spiliotis and McMurray, 2020). It is therefore not surprising that septins are involved in many important cellular processes, such as cytokinesis, phagocytosis, ciliogenesis, cytoskeletal dynamics during bacterial entrapment, barrier formation, cellular polarization and morphogenesis, besides several others that demand membrane remodeling and/or scaffolding capabilities (Longtine et al., 2000; Hu et al., 2010; Mostowy et al., 2010; Mostowy and Cossart, 2012; Ewers et al., 2014; Beber et al., 2019; Falk et al., 2019; Robertin and Mostowy, 2020; Szuba et al., 2021).

In order to remodel cell morphology, septins interact with other cytoskeletal elements, for example in the nucleation and branching of actin filaments (Hu et al., 2012; Mavrakis et al., 2014). However, although there are several reports in the literature of co-localization of septins with actin and microtubules, it is not yet fully understood how these interactions occur, whether they are direct or indirect and whether they depend on the polymerization of septins or not (Mavrakis et al., 2014; Spiliotis, 2018; Spiliotis and Nakos, 2021). A recent report assessing the self-oligomerization of budding yeast septins on biomimetic membranes showed that octamers (rather than full filaments) were able to reshape membranes (Vial et al., 2021).

As a cytoskeleton component, septins are proteins with the inherent ability to self-assemble into filaments (Byers and Goetsch, 1976; Field et al., 1996; Frazier et al., 1998; Sirajuddin et al., 2007), and subsequently into more sophisticated architectures, as reviewed by Marquardt et al. (2019). The molecular basis for this is still an outstanding research question. Of note is that many post-translational modifications such as phosphorylation, acetylation, ubiquitination and sumoylation have already been observed modulating septin filament dynamics (Johnson and Blobel, 1999; Takahashi et al., 1999; Zhang et al., 2000; Hernández-Rodríguez and Momany, 2012; Ribet et al., 2017). Additionally, septins bind (and often hydrolyse) GTP, justifying their inclusion as members of the diverse family of P-loop GTPases (Leipe et al., 2002; Weirich et al., 2008; Sirajuddin et al., 2009). At least in yeast, GTP hydrolysis appears to be involved in the assembly of specific heterocomplexes (Weems and McMurray, 2017), and evidence also suggests that the nature of the bound nucleotide may play a role in membrane association by higher-order septin assemblies (Bertin et al., 2010; Bridges et al., 2014). However, tracing a correspondence between yeast and mammalian septins is not trivial and hampered by the significant phylogenetic differences between the heterocomplexes observed in fungi and animals. Thus, the relationship between filament assembly and GTP hydrolysis requires further work in order to be fully understood.

Septins are ubiquitous in opisthokonts, but orthologous septin families are also present in a broader range of other eukaryotes (Nishihama et al., 2011). The number of genes coding for septins in different organisms is quite variable. For example, there are species with only one or two genes, such as *Chlamydomonas* and *Caenorhabditis elegans*, respectively. Conversely, extensive gene amplification in vertebrates has led to 13 septins genes in humans and mice. In more extreme cases*,* gene duplication has resulted in further paralogues for many septins, culminating in a set of at least 17 in *Danio rerio,* for example (Willis et al., 2016). In view of such expansion, mammalian septins (Kinoshita, 2003), and later metazoans (Cao et al., 2007), have been classified into four groups, based on sequence similarities: SEPT2 group (SEPT1, SEPT2, SEPT4 and SEPT5), SEPT3 group (SEPT3, SEPT9 and SEPT12), SEPT6 group (SEPT6, SEPT8, SEPT10, SEPT11 and SEPT14) and SEPT7 group (SEPT7 alone) (Pan et al., 2007; Hilary Russell and Hall, 2011).

The dynamics of septin filament assembly changes radically during the cell cycle, albeit in a highly regulated way, both in time and space (Marquardt et al., 2020). Several binding partners could be key players in this process in which they may act by regulating septin remodeling (Nakahira et al., 2010; Sandrock et al., 2011). Some of the regulatory proteins of the cytoskeleton which show direct or indirect association with septins in different organisms include anillin (Kinoshita et al., 2002), CDC42 effector proteins (CDC42EP or Borg’s) (Sheffield et al., 2003), end-binding protein 1 (EB1) (Nölke et al., 2016; Nakos et al., 2019a) along with many others. An exhaustive review of septin binding partners can be found in Neubauer and Zieger (2017).

Due to their wide-ranging roles in fundamental cellular processes, septin dysfunction has been implicated in a series of pathologies, including (but not limited to) male infertility, neurodegenerative diseases and cancer (Peterson and Petty, 2010; Angelis and Spiliotis, 2016; Palander et al., 2017). The regulation of septin expression is crucial for orchestrating cell homeostasis and thus it is not uncommon for pathologies, such as certain types of cancer, to be associated with changes in the levels of protein expression or mutations in a particular septin gene (Angelis and Spiliotis, 2016). The SEPT9_i1 isoform, for instance, was found to be overexpressed in breast tumors and linked to many other types of cancer (Gonzalez et al., 2009). Additionally, several neuropathies, such as Parkinson’s and Alzheimer’s diseases, have also been associated with septin accumulation (Kinoshita et al., 1998; Ageta-Ishihara et al., 2013; Tokhtaeva et al., 2015) and biophysical studies have demonstrated that *in vitro* individual septin subunits are unstable and tend to aggregate into amyloid-like structures (Garcia et al., 2007; Pissuti Damalio et al., 2012; Kumagai et al., 2019). This seems to imply that, under physiological conditions, heterocomplexes would be expected to be the predominant intracellular species, underlining their natural tendency to self-organize.

Although more than 50 years have passed since the discovery of septins, much remains to be learned about the potential roles of monomers, oligomers, filaments and higher-order structures. Taken together, data from structural studies of septins have brought important contributions to clarify these issues. In this review, it is not our intention to overload the reader with excessive structural detail. Instead, we aim to collate the major structural discoveries described over recent years, bring them together into a single document and relate them, where possible, to septin function. In so doing, we hope to stimulate the appearance of new hypotheses which will throw light on structure-function relationships at all levels of septin organization.

## 2 The Septin Domain Architecture

**FIGURE 1:**
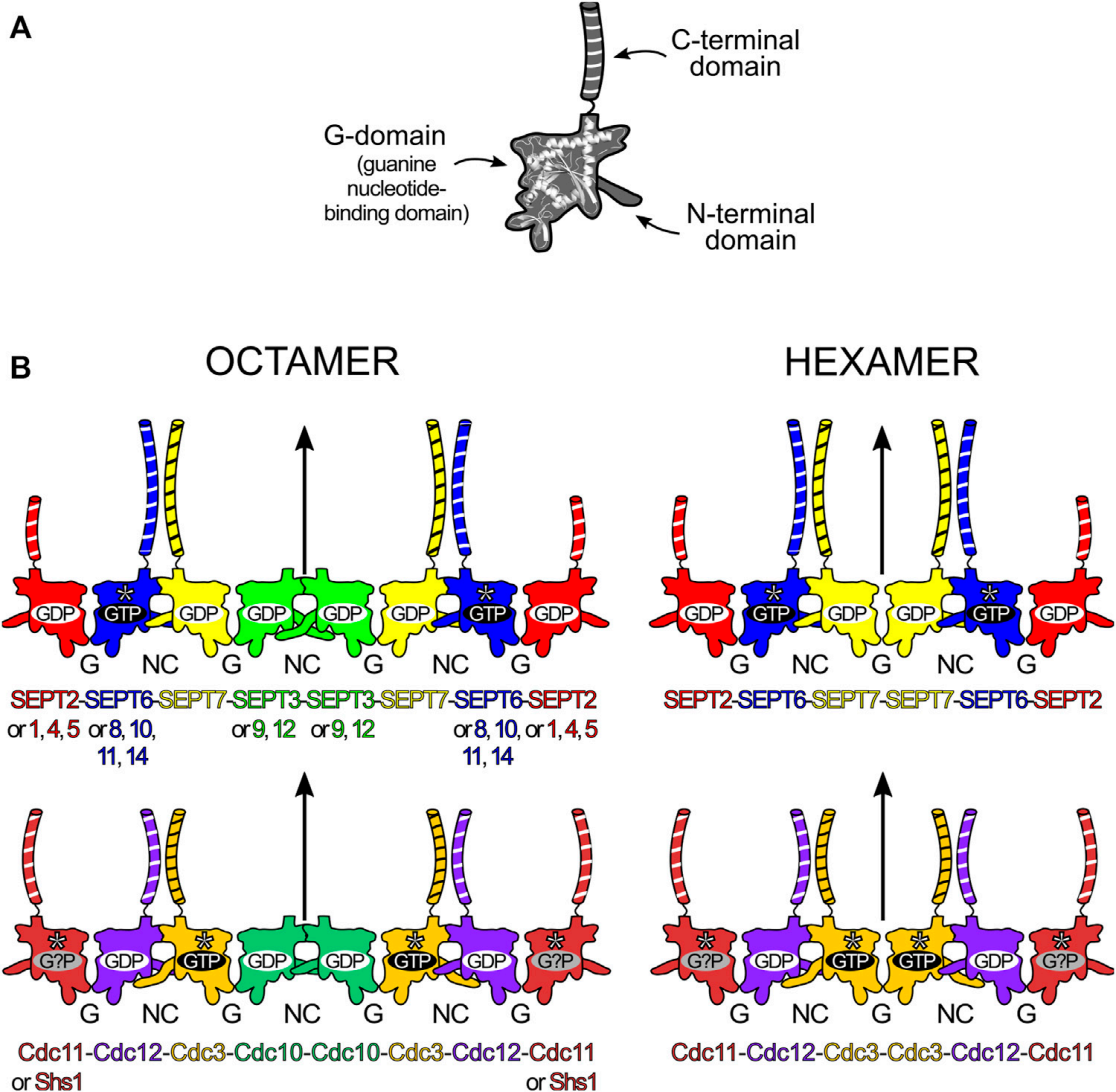
Structural elements and core particle composition of septin filaments. **(A)** The septin structure is divided into three main domains: the N-terminal domain, the guanine nucleotide-binding domain (or G-domain) and the C-terminal domain (depicted with black and white stripes). **(B)** Octameric **(left)** and hexameric **(right)** core particles in human **(top)** and baker’s yeast (*Saccharomyces cerevisiae*, **bottom**). In human septins, replacements according to “Kinoshita’s rule” are depicted on the octamer. Straight arrows represent the twofold rotational symmetry (diad) axis at the center of the core particle. The yeast hexameric oligomer presented is that formed in the absence of Cdc10 (Cdc10-less oligomer). SEPT6-group members (blue), Cdc3 and Cdc11 are catalytically inactive and do not hydrolyse GTP (asterisks). Cdc11 seems to bind only cytosolic GDP (although speculatively) and Shs1 can also cap octamers, replacing Cdc11. The long N-domains in SEPT3 and Cdc3 are also represented.

Septins belong to the family of small GTPases and are characterized by possessing a GTP-binding (G-) domain. This was identified in the first amino acid sequences of septins by the presence of the P-loop motif, characteristic of GTP/ATP binding proteins (Longtine et al., 1996). The GTPase activity itself was later demonstrated by *in vitro* assays (Field et al., 1996). Different from other small GTPases, septins possess variable N- and C-terminal extensions or domains (or simply N- and C-domains) (Figure 1A; Field and Kellogg, 1999). Although there is no exact definition (in terms of residue position) for the limits of each domain, the overall consensus is to accept that the N-domain lies upstream of the first β-strand of the characteristic G-domain fold, prior to the P-loop, and the C-domain lies downstream to the final α-helix (α6).

The N-domain is highly variable in both length and amino acid sequence and is predominantly unstructured, being classified as an IUD (intrinsically unstructured domain) (Garcia et al., 2006; Pan et al., 2007). At its C-terminus, the N-domain has a region which forms an α-helix, dubbed α0, which includes a polybasic sequence (PB1) associated with membrane phospholipid interaction (Zhang et al., 1999; Bertin et al., 2010). The G-domain is highly conserved and has all the necessary motifs for binding GTP. Among the motifs shared with small GTPases are the P-loop or Walker A box (Walker et al., 1982) (also called G1) which oversees coordinating the phosphate moieties of the nucleotide, the switches I and II (G2 and G3 respectively) which are related to the hydrolytic mechanism itself and the G4 motif that confers specificity for GTP over other nucleotide triphosphates (Pan et al., 2007). Four septin-specific motifs have been identified in the G-domain (Sep1-4) as well as six specific residues which are conserved in 86–94% of all septin sequences (Pan et al., 2007). The G-domain terminates in a characteristic sequence known as the septin unique element (SUE), important for filament formation (Versele et al., 2004). The C-domain is variable but typically includes a region compatible with the formation of coiled coils (Versele et al., 2004; Pan et al., 2007), although this is absent from some septins, including the SEPT3 group in humans and Cdc10 in yeast.

## 3 The Septin Hetero-Oligomer: The Building Block for Polymerization

All of the many important functions that septins perform in the cell are related to their ability to assemble into highly organized filaments. Although filaments can subsequently generate many kinds of higher-order structures (bundles, rings, gauzes, etc.), all are comprised of filaments, which are built by end-to-end association of core particles (also known as protofilaments or simply oligomers). A core particle is a nonpolar, linear hetero-oligomeric complex of septin subunits, interacting side-by-side, like beads on a string (Figure 1B). Septins use different domains for hetero-oligomerization: with each monomer interacting with its neighbors by alternate interfaces, named NC (from the N- and C-terminal domains) and G (from the G-domain) (Figure 1B). Each domain and interface will be discussed in further detail later in this review.

In mammals, both *in vitro* and *in vivo*, septins from the different groups assemble to form the core particles. These are symmetric and have 2*n* subunits forming a palindromic arrangement, with *n* being the number of different septins in its composition. This results in a diad axis (*C*_2_) at its centre, lying perpendicular to the main axis of the oligomer (Figure 1B). A septin filament is then likely assembled *in vivo* by the end-to-end annealing/collision of these core particles on the plasma membrane (Bridges et al., 2014). The core particle is, therefore, the building block of the filament and is usually defined as the oligomer which persists in high ionic strength solution *in vitro*, as several studies have shown how salt concentration modulates filament polymerization (Frazier et al., 1998; Versele et al., 2004; Bertin et al., 2008).

The number of septin monomers in the core particle can be variable and is species-dependent. For example, *C. elegans* has only two septins (Unc-59 and Unc-61) which form tetramers and polymerize to play roles in cytokinesis, migration and cell polarity (Nguyen et al., 2000; John et al., 2007). In *S. cerevisiae*, baker’s yeast, four septins (Cdc3, Cdc10, Cdc11/Shs1 and Cdc12) generate octamers (Bertin et al., 2008; Garcia et al., 2011). In mammalian cells, septins from three or four different groups can be incorporated into the core particle, leading to the formation of hetero-oligomeric hexamers or octamers, respectively (Kim et al., 2011; Sellin et al., 2011). A noteworthy feature, usually referred to as “Kinoshita’s rule” (Valadares et al., 2017; Spiliotis and McMurray, 2020), is that within the core particle, each septin is predicted to be interchangeable with another from the same group, thereby generating diversity (Kinoshita, 2003). On the other hand, as far as is known, the position of each septin group within the particle is fixed (Mendonça et al., 2019). Kinoshita’s rule predicts 20 and 60 different combinations for hexamers and octamers respectively. How many of these are physiologically relevant and how their properties and functions may vary represent important outstanding questions.

In humans, the difference between the hexamer and the octamer is the incorporation of a SEPT3-group member in the latter which is absent from the former (Füchtbauer et al., 2011; Kim et al., 2011; Sellin et al., 2011; Sellin et al., 2014; Abbey et al., 2016). For many years, the subunit order of the human hexamer (and therefore that implied for the octamer) was erroneously considered to be SEPT7-6-2-2-6-7. New studies have shown the correct order to be SEPT2-6-7-7-6-2 (Mendonça et al., 2019; Mendonça et al., 2021) or SEPT2-6-7-3-3-7-6-2 in the case of octamers (Figure 1B; DeRose et al., 2020; Soroor et al., 2021; Iv et al., 2021).

Recent publications from *in vitro* experiments using mammalian septins bring fresh data to support the idea of hexamers and octamers coexisting within a single filament (DeRose et al., 2020; Soroor et al., 2021). This appears completely plausible in the light of the corrected subunit order since both hexamers and octamers have an exposed SEPT2 NC-interface at their termini (DeRose et al., 2020; Soroor et al., 2021), raising the possibility of a wide range of filaments differing in their hexamer-to-octamer ratio. There is also evidence pointing towards specific cellular functions that only arrangements including SEPT3-group members could perform (Estey et al., 2010; Bai et al., 2013; Kuo et al., 2015; Karasmanis et al., 2018).

In mitotic yeast cells, the octameric core particle is Cdc11-12-3-10-10-3-12-11 (Figure 1B; Versele et al., 2004; Bertin et al., 2008). Shs1, a non-essential septin in *S. cerevisiae*, can substitute for Cdc11, usually giving rise to ring-like arrangements (Garcia et al., 2011). Octamers polymerize through the NC-interface of Cdc11 (or Shs1), matching that which occurs in human septins; but in *C. elegans*, the Unc septin oligomers (Unc-59-61-61-59) seem to polymerize through an exposed G-interface (John et al., 2007). Yeast septins can also form hexamers when specific subunits are absent (Cdc10-less oligomers, Cdc11-12-3-3-12-11 and Cdc11/Shs1-less oligomers, Cdc12-3-10-10-3-12), but these are unable to form long filaments (Frazier et al., 1998; Versele et al., 2004; McMurray et al., 2011; Johnson et al., 2020). Albeit considered rare in fungi, naturally-occurring septin hexamers have been found in *Aspergillus nidulans* (oligomers lacking AspD, a Cdc10 homologue), where they coexisted with octamers (Hernández-Rodríguez et al., 2014).

The GTP binding and hydrolytic ability of each septin are likely related to the order and overall composition of the assembly (Sirajuddin et al., 2009; Weems and McMurray, 2017; Abbey et al., 2019). Some septins display no (or very little) GTPase activity as they lack the catalytic threonine from the G2 motif in switch I. This is the case for all SEPT6-group septins (Sirajuddin et al., 2007; Zent and Wittinghofer, 2014) and both Cdc3 and Cdc11 in yeast (Versele and Thorner, 2004). It is intriguing, however, that the non-catalytic septins in mammals and yeast do not map to equivalent positions within the core particles (Figure 1B). Furthermore, in yeast, one of the catalytically inactive subunits (Cdc3) is bound to GTP, as anticipated, while the other (Cdc11) is hypothetically bound to GDP, presumably acquired from the cytosol (Farkasovsky et al., 2005; Weems and McMurray, 2017). However, another model claims that Cdc3 and Cdc11 are apoproteins even when incorporated into octamers (Baur et al., 2019). This conundrum is expected to be resolved once more structural information on yeast septins becomes available.

## 4 The Current Completeness of the Structural Information Available

Back in 2007, the first hetero-oligomeric septin structure was reported. This was the mammalian SEPT2-6-7 hexamer and was solved by X-ray diffraction at 4.0 Å resolution (Sirajuddin et al., 2007). For many years, this was the only structural model available for a hetero-oligomeric complex and due to its low resolution, many questions were left unanswered. Some of these were clarified by employing a “divide-and-conquer” strategy, first by the structure determination of single septin G-domains (Sirajuddin et al., 2007; Sirajuddin et al., 2009; Serrão et al., 2011; Zent et al., 2011; Macedo et al., 2013; Zeraik et al., 2014; Brognara et al., 2019) and later by better understanding the interfaces formed between them by solving the structures of both homo- and heterodimeric complexes (Brognara et al., 2019; Castro et al., 2020; Rosa et al., 2020). Recently, the original hexameric complex, SEPT2-6-7, has been solved by cryo-EM at 3.6 Å resolution, providing important additional information (Mendonça et al., 2021).

**FIGURE 2:**
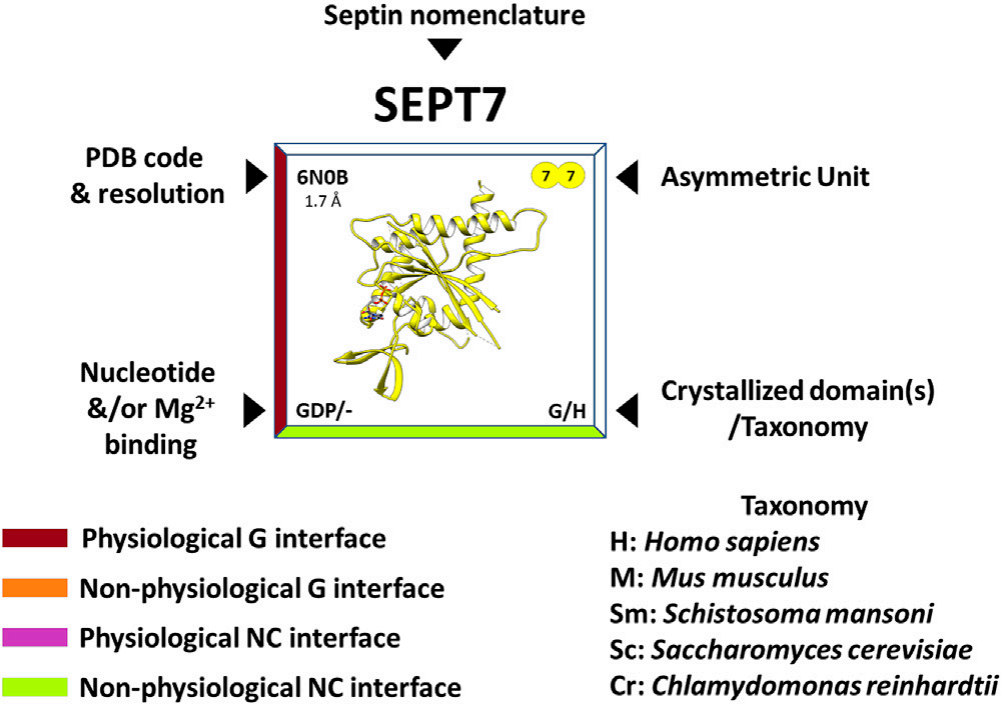
Features of the “Septin Chart” (see complete figure in Supplementary Figure S1). The PDB code and resolution are shown in the upper left corner, the contents of the asymmetric unit in the upper right, the presence and nature of the bound nucleotide/magnesium in the lower left corner and finally, in the lower right, the purified and crystallized domain(s) as well as the acronym of the organism to which it belongs (e.g., H = *Homo sapiens*). In addition, the edges of the box surrounding the structure are color-coded and refer to the contact interfaces observed in the crystal: burgundy for a physiological G-interface, orange for a non-physiological G-interface, purple for a physiological NC-interface and lime for a non-physiological NC-interface. In some cases, in order to observe some of the interfaces, it is necessary to apply crystallographic symmetry operations to the asymmetric unit.

Nowadays, the richness of the structural information available (mainly from human septins) has made it possible to rationalize many functional aspects of the individual domains and the structural motifs they contain. At the time of writing, there are 32 septin structures available in the PDB, representing different domains and oligomeric states. In the “Septin Chart” (Supplementary Figure S1), we present this structural diversity, in a format inspired by the periodic table, to facilitate access to basic structural information (the asymmetric unit, PDB code, resolution, bound nucleotides, etc.). As a representative example, Figure 2 shows the septin structure with the highest resolution currently available (SEPT7, PDB:6N0B). Each cell in the table explicitly indicates if the interfaces observed in the crystal structure are expected to be physiological (based on the canonical model of Figure 1B) or non-physiological (*promiscuous*). The latter are frequently observed in crystal structures and raise the intriguing question of why they apparently do not form physiologically.

## 5 The G-Domains

**FIGURE 3:**
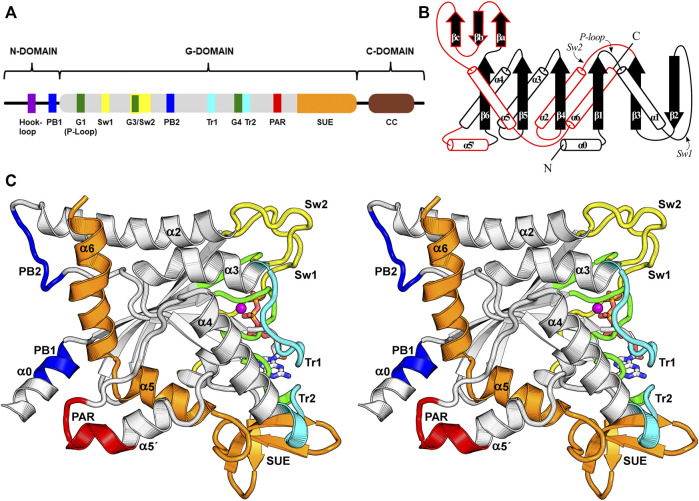
The septin G-domain. **(A)** Schematic representation of the septin domains and their hallmark features. The N-domain presents variable length and encompasses the α0 helix, which contains the polybasic region 1 (PB1). The G-domain contains all the essential nucleotide-binding motifs (G1, G3, G4) and the switches important for catalysis. Other functional elements related to interface formation, such as a second polybasic region (PB2), a polyacidic region (PAR), the *trans*-loops 1 and 2 (Tr1 and Tr2) and the septin unique element (SUE) are also indicated. The C-domain often contains heptad repeats that form coiled coils (CC). **(B)** A schematic topology diagram for the septin G-domain fold. Three unique features observed in septins are highlighted in red. **(C)** Stereo representation of the G-domain highlighting some of its features employing the same color code used in panel **(A)**. Several of these features and secondary structure elements are labeled. The PB1, PB2 and PAR are close to the NC-interface, while the switch I (Sw1), switch II (Sw2), Tr1 and Tr2 participate in the G-interface. The SUE is an exclusive feature of septins and participates in both interfaces. The Mg^2+^ ion is coloured in magenta.

In the following section, we describe the “anatomy” of the G-domain, and its structural components (motifs), principally those for which it has been possible to ascribe a specific function. Figure 3 depicts their spatial disposition and also establishes the standard nomenclature employed for the elements of secondary structure which characterize the septin fold (Valadares et al., 2017). However, in this review, whilst we preserve the standard names of the six strands which comprise the main β-sheet (β1-β6), we propose that those of the three-stranded β-meander should be renamed βa, βb and βc, rather than β9, β10/7, and β8, for the sake of simplicity. Additionally, we make use of a new nomenclature to refer to particularly important positions/residues employing the following format: “residue(motif)”. For example, Thr(Sw1) refers to the catalytic threonine from switch I. This nomenclature eliminates the need for quoting specific residue positions, which vary from septin to septin. The nomenclature can be extended to the use of “residue (motif/group)” in order to indicate a specific septin group. This is particularly useful when referring to the so called *characteristic* residues (amino acid residues present essentially in a unique septin group and absent from all others) (Rosa et al., 2020). The new labels can be readily converted to the numerical format by using Supplementary Table S1 (human septins) and Supplementary Table S2 (septins from other organisms).

The G-domain is the most highly conserved among septins and is also generally the longest, although the longest isoform of SEPT9 has an N-domain of comparable size. Its fold resembles that of Ras GTP-binding proteins (Pai et al., 1989; Pai et al., 1990), displaying a central six-stranded β-sheet enclosed by α-helices in an αβα sandwich. However, when compared to Ras, septins display three additional exclusive features that are linked to their functions (highlighted in red on Figure 3B).

**FIGURE 4:**
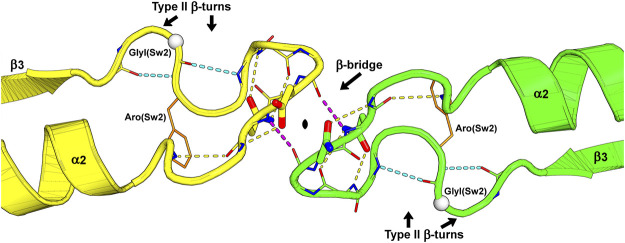
The switch II region at the G-interface of the SEPT7-SEPT3 heterodimer (PDB:6UQQ). An essentially identical arrangement is observed at all physiological G-interfaces. The wide-type β-bridge, which lies on the pseudo-twofold symmetry axis relating the two monomers, is labeled, and its two hydrogen bonds are colored in purple. Over the β-bridge, an aspartic acid in SEPT7 (yellow) and an asparagine in SEPT3 (green), i.e., the positions Asx(Sw2), are represented as sticks and positioned on opposite sides of the symmetry axis (black lozenge at the center). Under the β-bridge, each subunit presents an asparagine residue, Asn(Sw2), whose side chain is represented as sticks, forming hydrogen bonds to both the main chain nitrogen and oxygen atoms of the residue three positions prior in the sequence. In each subunit, two consecutive type II β-turns forming an “S” shape are labeled, and their hydrogen bonds are colored in cyan. Some side chains and main chain atoms were removed for clarity. Aro(Sw2) is indicated as it may play a role in communication between adjacent interfaces and GlyI(Sw2) is represented as a white sphere.

The first septin exclusive feature encompasses the longer switch II region (which participates in G-interface dimerization, Figure 4), an elongated α2 helix spanning the G- and NC-interfaces, and a lengthy loop containing the second polybasic region (PB2) that plays a critical role at the NC-interface (see Section 9). This long loop connects α2 to β4 and contains part of the highly conserved septin-specific motifs, Sep1 (ExxxxR) and Sep2 (DxRV/IHxxxY/FFI/LxP) (Pan et al., 2007). The loop runs underneath α2, preventing it from making direct contact with the underlying β-sheet and giving it structural autonomy (Castro et al., 2020). In total, this first feature adds 28 residues to human septins when compared to the equivalent region in H-Ras p21 (Pai et al., 1989; Pai et al., 1990).

A second feature unique to septins is an extended loop followed by an additional helix, termed α5′. These elements connect the α4 helix to the β6 strand, and correspond to at least 20 additional residues when compared to Ras. This region, and markedly the α5′ helix, displays low sequence conservation, except for a polyacidic region (PAR) located at the end of the loop and the beginning of α5′. The PAR participates in the NC-interface as a multipurpose element: in some structures it is observed interacting with the PB1 of the α0 helix (Figure 3C), and in others, it interacts with the PB2 that succeeds the α2 helix (see Section 9 and Section 10.1).

The final distinct feature is the septin unique element (SUE) (Versele et al., 2004), which also spans from the G-to the NC-interface and enables filament formation. The SUE is roughly 60 residues in length and may be divided into two portions. The first half comprises three small β-strands that form a very twisted β-meander (βa-βc). This region is an integral part of the G-interface, contacts the nucleotide and has a significant role in G-interface dimerization. A single mutation T282Y in the SEPT3 β-meander favors the formation of homodimeric G-interfaces in solution, in contrast to monomers formed by the wild type (Macedo et al., 2013). This mutation was also necessary to stabilize the SEPT7-SEPT3 heterodimeric complex for crystallographic studies (Rosa et al., 2020). The second part of the SUE begins with two consecutive turns formed mostly by conserved residues, and continues through helices α5 and α6, the latter being the second longest helix in the G-domain, forming part of the NC-interface. Helices α5 and α6 together form an elbow-like structure which has recently been shown to be important for stabilizing helix α0 from the neighboring subunit within the interface (see Section 9). The SUE is, therefore, an integral part of both interfaces and explains why septins, unlike other small GTPases, are able to polymerize.

### 5.1 The Fundamental Elements of the G-Domain

#### 5.1.1 Switch I

**FIGURE 5:**
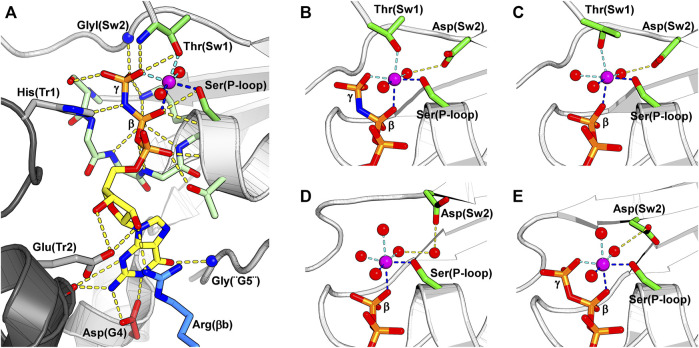
The nucleotide-binding pocket and the different modes of magnesium coordination. The β-phosphate, Ser(P-loop) and a water molecule participate in all depicted Mg^2+^ coordination schemes, and their coordination to the Mg^2+^ is shown by dark blue dotted lines. The magnesium ion is colored in purple. **(A)** A GTP analogue (GMPPNP) bound to SEPT12 (PDB:6MQ9). The nucleotide and the residues interacting with it are represented as sticks. Key residues from the septin versions of the five classical nucleotide-binding elements found in small GTPases are labeled: G1 is represented by Ser(P-loop), G2 by Thr(Sw1), G3 by GlyI(Sw2), G4 by Asp(G4) and G5 by Gly(“G5”). Often septins are said not to possess G2 and G5, but here we describe their remnants explicitly as such. The conserved arginine from the SUE, Arg(βb), is colored in blue. On the left side of the figure, two residues from the other subunit of the G-interface (dark grey) are represented; a histidine from the *trans*-loop 1, His(Tr1), that binds to the β-phosphate, and a glutamate from the *trans*-loop 2, Glu(Tr2), that forms a hydrogen bond to the ribose ring. **(B)** Magnesium coordination in a catalytically active septin bound to the non-hydrolysable GTP analogue, GMPPNP (PDB:6MQ9). **(C)** A *tightly* bound Mg^2+^ in a catalytically active septin bound to GDP. Thr(Sw1) remains as a ligand in this case, and Asp(Sw2) continues to coordinate the metal via a water molecule (PDB:6MQK). **(D)** In a *weakly* bound Mg^2+^ ion, also in the presence of GDP, Thr(Sw1) has now been replaced by a water molecule, and the participation of Asp(Sw2) is mediated by two waters rather than one (PDB:6N12). **(E)** Magnesium coordination in a non-catalytic septin bound to GTP. The Mg^2+^ coordination sphere is formed by the β- and γ- phosphates, Ser(P-loop) and three water molecules, one of which is held by the side chain of Asp(Sw2) (PDB:6UPQ).

The switch I is a long solvent-exposed loop connecting helix α1 to strand β2. It inherits its name from the small GTPases, in which switch I is part of the universal loaded spring mechanism that promotes GTP hydrolysis conformation (Vetter and Wittinghofer, 2001). A threonine residue from switch I, Thr(Sw1), is essential for the hydrolytic mechanism (Sirajuddin et al., 2009), and its main chain nitrogen atom is observed interacting with the γ-phosphate in structures of catalytic septins bound to GTP analogues, while its side chain coordinates the Mg^2+^ ion and its main chain carbonyl, the catalytic water (Figure 5A).

Switch I is arguably the least conserved region of the G-domain in human septins. The region is longer than in H-Ras, and is incomplete in many crystal structures. Its conformation depends on the septin group, the bound nucleotide, crystal packing and whether it forms a G-interface with a physiological partner or not. This conformational flexibility is physiologically relevant because in septins switch I has an additional role in the formation of the G-interface and may hold the key to selecting the correct G-interface partner (see Section 6.2) (Rosa et al., 2020).

#### 5.1.2 Switch II

Switch II both participates in the hydrolytic mechanism and contributes to G-interface stability. It contains the G3 motif, an integral part of the universal switch mechanism. This conserved motif contains a glycine residue, GlyI(Sw2) in Figure 5A, that binds to the γ-phosphate and an aspartic acid residue, Asp(Sw2) in Figures 5B–E, that coordinates the Mg^2+^ ion via a water molecule. After hydrolysis, this region adopts a slightly different conformation, positioning GlyI(Sw2) at the centre of a planar S-shaped structure formed by two consecutive type II β-turns (Figure 4). Following GlyI(Sw2) is an aromatic residue, Aro(Sw2), that is able to adopt different rotamers and it has been suggested that this may play a role in communication between adjacent G- and NC-interfaces (see Section 10.2).

#### 5.1.3 Helix α2

In septins, helix α2 is twice the size of its equivalent in small GTPases and extends from the G-to the NC-interface. It connects switch II at the G-interface to PB2 at the NC-interface, two regions that display a degree of conformational plasticity. These traits flag α2 as a candidate to act as a conduit for information transfer between the G and NC-interfaces (see Section 10.2).

#### 5.1.4 The Polyacidic Region and Alpha Helix 5′

All septins present a polyacidic region (PAR) at the beginning of helix α5′ and the loop preceding it. The angle of helix α5′ in relation to the G-domain varies which may influence the position of the PAR with respect to the PB1 of helix α0 from the neighboring subunit when the NC-interface is in its canonical (open) conformation (see Section 9 and Section 10.1). Indeed, the cryo-EM structure of the SEPT2/6/7 complex confirms a direct interaction between the PAR and PB1, when α0 is stored within the interface (Figure 3C).

#### 5.1.5 The Septin Unique Element and Alpha Helix 6

The septin unique element (SUE) (Versele et al., 2004) is a continuous sequence which forms the C-terminal region of the G-domain and, as its name implies, is conserved, essential and exclusive to septins. It contributes to both interfaces as described above.

Helix α6 is the final element of secondary structure to form part of the G-domain and lies perpendicular to the main filament axis at the NC-interface. It also lies perpendicular to helix α2, and together these long helices stand out as a distinctive feature of the G-domain. A characteristic feature of α6 is a visible unwinding of the helix at its center, generating an α-aneurism (Keefe et al., 1993). The conserved nature of the aneurism and its conspicuous location at the NC-interface suggests that it may have a functional role. However, this has yet to be elucidated. Intriguingly, due to a residue deletion, the single septin from *Chlamydomonas reinhardtii* does not present the α-aneurism resulting in significant topographic alterations to the NC-interface which is not observed in the crystal structure thereby impeding the *in crystallo* formation of filaments (Pinto et al., 2017). It is, therefore, unclear if this single septin is able to form homofilaments *in vivo.*

Helix α6 presents two conserved charged residues, glutamic acid Glu(α6) and arginine Arg(α6), that take part in an extensive hydrogen-bonding network which forms the upper part of the NC-interface. The pattern of salt bridges depends on the conformation of the NC-interface (see Section 9.1).

### 5.2 The GTP-Binding Site and the Magnesium Coordination States

#### 5.2.1 Classic Nucleotide-Binding Motifs

All currently deposited structural data on septins (with the exception of Cdc11, PDB:5AR1) exhibit a bound guanine nucleotide as an integral part of the G-domain, where it participates in the G-interface. Small GTPases employ five classic motifs (G1-G5) in nucleotide binding, and septins use their versions of these motifs to maintain the nucleotide tightly bound and to perform catalysis.

The G1 motif presents the consensus GxxGxGKS/T and forms the P-loop. Several main chain nitrogen atoms of this motif interact with the β-phosphate, while the side chain of Ser(P-loop) coordinates the Mg^2+^ ion. This region is followed by a threonine residue in helix α1 that binds the α-phosphate. Switch I (Sw1) includes the septin version of the G2 motif, which contributes a threonine residue, Thr(Sw1), that binds both the γ-phosphate and the Mg^2+^ ion and is essential for catalysis (Sirajuddin et al., 2009). The G3 motif is part of switch II (Sw2) where, in catalytic septins, the glycine residue GlyI(Sw2) binds the γ-phosphate (Figure 5A). Moreover, this region bears an aspartic acid residue, Asp(Sw2), that binds and orients a water molecule to coordinate the Mg^2+^ ion (Figures 5B–E **)**. Oddly, in catalytically inactive septins, Asp(Sw2) may be replaced by a serine, an asparagine or a glutamate, the latter being able to coordinate the Mg^2+^ ion directly. In the G4 motif (A/GK/RAD in human septins), the lysine (or arginine) interacts with the ribose, while the aspartate (Asp(G4)) forms two hydrogen bonds to the guanine base. In septins, a single glycine, Gly(“G5”), represents the remnants of the G5 motif. Finally, human septins boast a conserved arginine residue in the septin unique element, Arg(βb) in Figure 5A, that is part of the nucleotide-binding pocket.

In addition to the residues from the G-domain to which the nucleotide is bound, two residues from the neighboring subunit reach across the G-interface and assist in nucleotide binding: a histidine from the *trans*-loop 1 (His(Tr1)) interacts with the β-phosphate and a glutamate from the *trans*-loop 2 (Glu(Tr2)) forms a hydrogen bond to the ribose (Figure 5A). Exclusive to algal septins, an arginine residue from the Sep3 motif interacts directly with the γ-phosphate of the neighboring subunit, acting like a catalytic “arginine finger” and accelerating GTP hydrolysis (Pinto et al., 2017).

#### 5.2.2 Magnesium Coordination States

Several coordination states for Mg^2+^ have been observed in different crystal structures (Figures 5B–E), and in approximately half the cases, the Mg^2+^ ion is either absent or cannot be modeled. A common feature of all such coordination schemes is the participation of Ser(P-loop), the β-phosphate and a water molecule.

In catalytically active septins, prior to catalysis, the Mg^2+^ ion is hexa-coordinated by a water molecule, Ser(P-loop), Thr(Sw1), the β- and γ- phosphates and Asp(Sw2) via a second water. After catalysis, however, GDP-bound septins display two possible magnesium coordination schemes. The first presents a *tightly* bound Mg^2+^, quite similar to that observed for GTP, but with a third water molecule replacing the γ-phosphate (Figure 5C). The second possibility is a *weakly* bound Mg^2+^ ion where a fourth water molecule replaces Thr(Sw1) and there are now two intervening waters between Asp(Sw2) and the metal. The transition from *tight* to *weak* binding may represent snapshots of different steps in the process of metal release upon catalysis.

In catalytic septins, switch II and particularly switch I are observed in very different conformations depending on the nucleotide bound, indicating that catalysis triggers major conformational changes at the G-interface. It would seem that the Mg^2+^ ion and its ligands are essential components of this mechanism. However, the full details of the role which Mg^2+^ plays during GTP binding and hydrolysis in the case of septins has yet to be fully elucidated.

## 6 The G-Interface

### 6.1 G-Interface Components and the Basis for Kinoshita’s Rule

With the first description of a septin structure (Sirajuddin et al., 2007), the critical role of the G-interface for the molecular assembly of the core particle became evident. As a result of the dozens of structures determined subsequently, the nature of the interactions involved at the interface has become clear and the exquisite manner by which stability, specificity and exchangeability arise have begun to be understood (Brognara et al., 2019; Rosa et al., 2020). In general, the G-interface is extremely well conserved in different structures, preserving almost perfectly the relative positions of the two monomers involved. Furthermore, no crystal structure presenting an intact G-interface has been observed in the absence of bound nucleotide. This suggests the ligand to be an integral component of the interface and/or that substrate hydrolysis through a GAP-like mechanism depends on dimer formation (Gasper et al., 2009; Sirajuddin et al., 2009; Zent and Wittinghofer, 2014). This section will describe the surface regions on the monomers which participate in the G-interface, highlighting the structural differences between groups and specifying the details which contribute to correct filament assembly.

**FIGURE 6:**
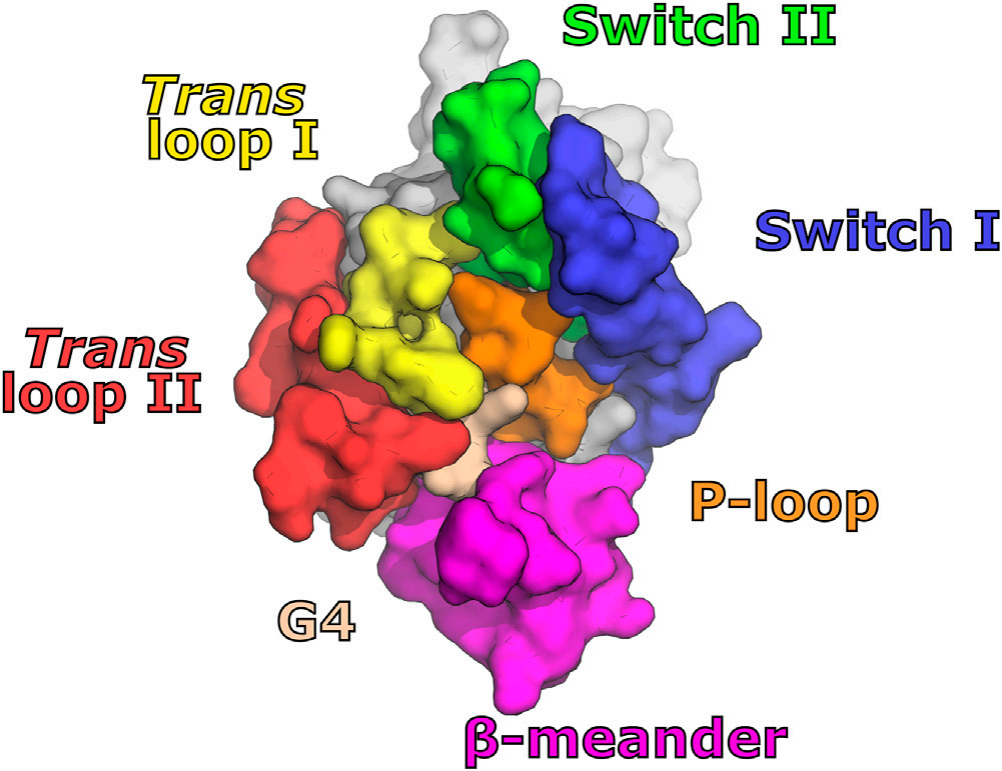
Motifs interacting at septin G-interfaces. Seven main contact regions are indicated on the G-side of the monomer. They are distributed in a diamond-like shape with switch II at the top; switch I, *trans*-loops 1 and 2, the P-loop and G4 at the center; and the β-meander at the bottom.

The standard model for the human hexamer implies the existence of G-interfaces between SEPT2 and SEPT6 and between two copies of SEPT7 (Figure 1B). In the octamer, the latter is replaced by a SEPT7-SEPT3 interface. At these interfaces, up to seven contact regions within the G-domain of each monomer form a diamond-like shape (Figure 6). At the top of the diamond, the two switch II regions interact, as shown in Figure 4. The central region, where the contact surface is widest, involves Tr1, the P-loop and G4. This also includes the switch I region in the case of SEPT2-and SEPT6-group member interfaces. At the bottom, the β-meander participates in interactions with Tr2 of the corresponding partner. Even at *promiscuous* interfaces, many of these features are preserved, including those involved in nucleotide binding. Although some subtle variation has been observed within the interface core (e.g., mutation to Glu(P-loop) in the SEPT3 group and in *Drosophila* Pnut/SEPT7 or to the β-meander in the SEPT3 group), it is the region at the rim of the interface, particularly switch I, which presents the greatest variability between septin groups (Rosa et al., 2020).

### 6.2 Switch I Determines the Selectivity at the G-Interface Formed Between Septins of the SEPT2 and SEPT6 Groups

**FIGURE 7:**
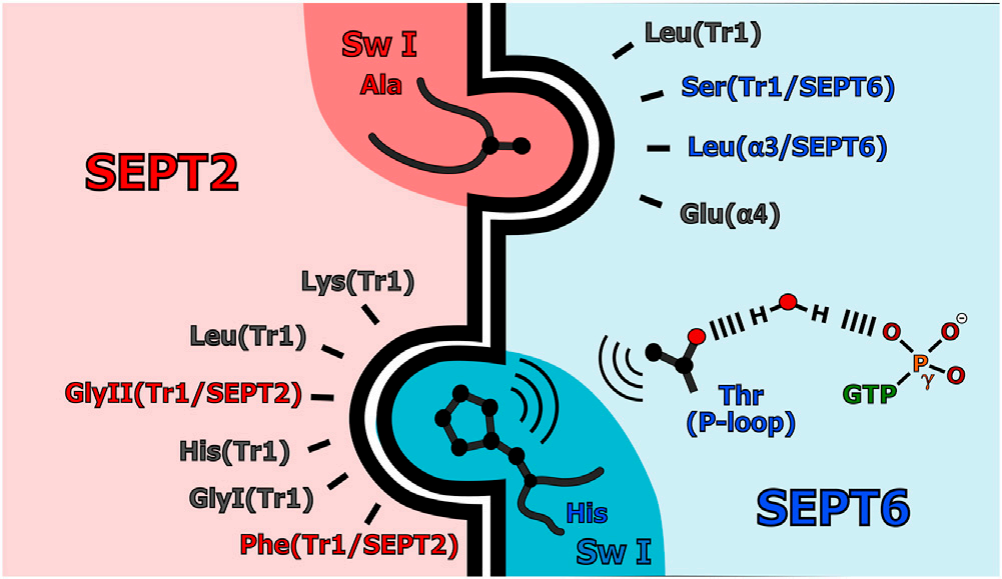
Interaction specificities in the SEPT2-SEPT6 G-interface. Simplified scheme for the heterotypic interface highlighting the importance of residues coming from switch I on both sides of the interface. Group-specific (*characteristic*) residues are shown colored, in red for SEPT2 and in blue for SEPT6. The network of interactions leading from the interface to the γ-phosphate of the GTP is shown.

How do the correct pairings of septins arise at the different G-interfaces of the core particle during spontaneous assembly? This is a question relating to the interactions involved in molecular recognition. Switch I makes a significant contribution to the contact area at the G-interface between septins of the SEPT2 and SEPT6 groups (but not those between SEPT7 and SEPT3) (Rosa et al., 2020). This raises the possibility that the selectivity that drives members of these two groups together may be related to selective interactions involving group-specific residues found within the switch I regions. Such interactions are shown in Figure 7 and Supplementary Figure S2.

An interaction network involving group-specific (or *characteristic*) residues is observed between the Tr1 of SEPT2 and the P-loop/Switch I of SEPT6/8/11 (Figure 7). The hydroxyl of a *characteristic* threonine residue in the SEPT6 group, Thr(P-loop/SEPT6), interacts with the γ-phosphate of GTP via a water molecule, thereby orienting its methyl group towards His(Sw1/SEPT6). This allows the correct orientation of the imidazole ring of His(Sw1/SEPT6) to accept a hydrogen bond from the main chain of SEPT2. His(Sw1/SEPT6) fits snugly into a pocket formed by Tr1 of SEPT2, whose conformation is determined by the *characteristic* residues Phe(Tr1/SEPT2) and GlyII(Tr1/SEPT2). Compared with septins from other groups, the *trans*-loop 1 of SEPT2 adopts a more extended conformation (Supplementary Figure S2A; Rosa et al., 2020). Both the ordering of switch I in SEPT6/8/11 and the conformation of *trans*-loop 1 in SEPT2 are necessary for correct partner pairing at the interface. No other group combination would provide the appropriate structural features for the fit schematically represented in Figure 7. This can be verified by observing the disordered and/or incomplete switch I regions, present at *promiscuous* G-interfaces such as the homodimer of SEPT2, *Sm*SEPT10 and SEPT3 (Sirajuddin et al., 2007; Zent et al., 2011; Macedo et al., 2013; Zeraik et al., 2014).

The involvement of GTP in the interaction network (Figure 7) suggests a functional significance for the lack of catalytic activity in the SEPT6 group. The persisting γ-phosphate should be considered a characteristic feature of the SEPT6 group as it aids in correctly orienting Thr(P-loop/SEPT6) towards His(Sw1/SEPT6). In the remaining groups of septins, this threonine is replaced by a serine, incapable of forming the hydrophobic contact with His(Sw1/SEPT6).

On the other side of the G-interface, in the switch I region of SEPT2, a *characteristic* amino acid Ala(Sw1/SEPT2), fits into a cavity in SEPT6/8/11 (Figure 7 and Supplementary Figure S2B). The presence of amino acids with longer side chains at this position in the SEPT3 group, or a proline in SEPT7 or even its absence altogether in the SEPT6 group (Supplementary Table S1) suggests that this interaction would only be viable in the case of the SEPT2 group (Rosa et al., 2020). Ala(Sw1/SEPT2) interacts via Van der Waals contacts with Ser(Tr1/SEPT6) which forms a hydrogen bond with the main chain of an aspartic acid in SEPT2. This interaction is only possible for the SEPT6 group where Ser(Tr1/SEPT6) is always either serine or threonine. This allows for the correct orientation of switch I of SEPT2, permitting Ala(Sw1/SEPT2) to fit into its complementary pocket in SEPT6.

In summary, the presence of *characteristic* residues converges on the correct structural organization of switch I and the *trans*-loop 1 of the SEPT6 and SEPT2 groups, favoring the formation of the specific G-interface. Since the interactions involved, by definition, can be generated by any member of the groups involved, this provides a molecular basis for the understanding of Kinoshita’s rule (substitutability between members within a group). As a “side effect”, we begin to understand the reason why the SEPT6 group lacks catalytic activity. By retaining the γ-phosphate, a network of interactions can form, guaranteeing the correct pairing of members of these two groups at the G-interface.

### 6.3 Switch II Interaction at Physiological G-Interfaces

The crystal structures of homodimers and heterodimers have made it possible to demonstrate that the complete ordering of switch II is related to the formation of physiological G-interfaces (Zent et al., 2011; Zeraik et al., 2014; Brognara et al., 2019; Castro et al., 2020; Rosa et al., 2020). The structure which results from the pairing of the two switch II regions is shown in Figure 4. In general, this pairing does not arise at *promiscuous* G-interfaces, which frequently appear in crystal structures of isolated G-domains of a single septin (Supplementary Figure S3).

**FIGURE 8:**
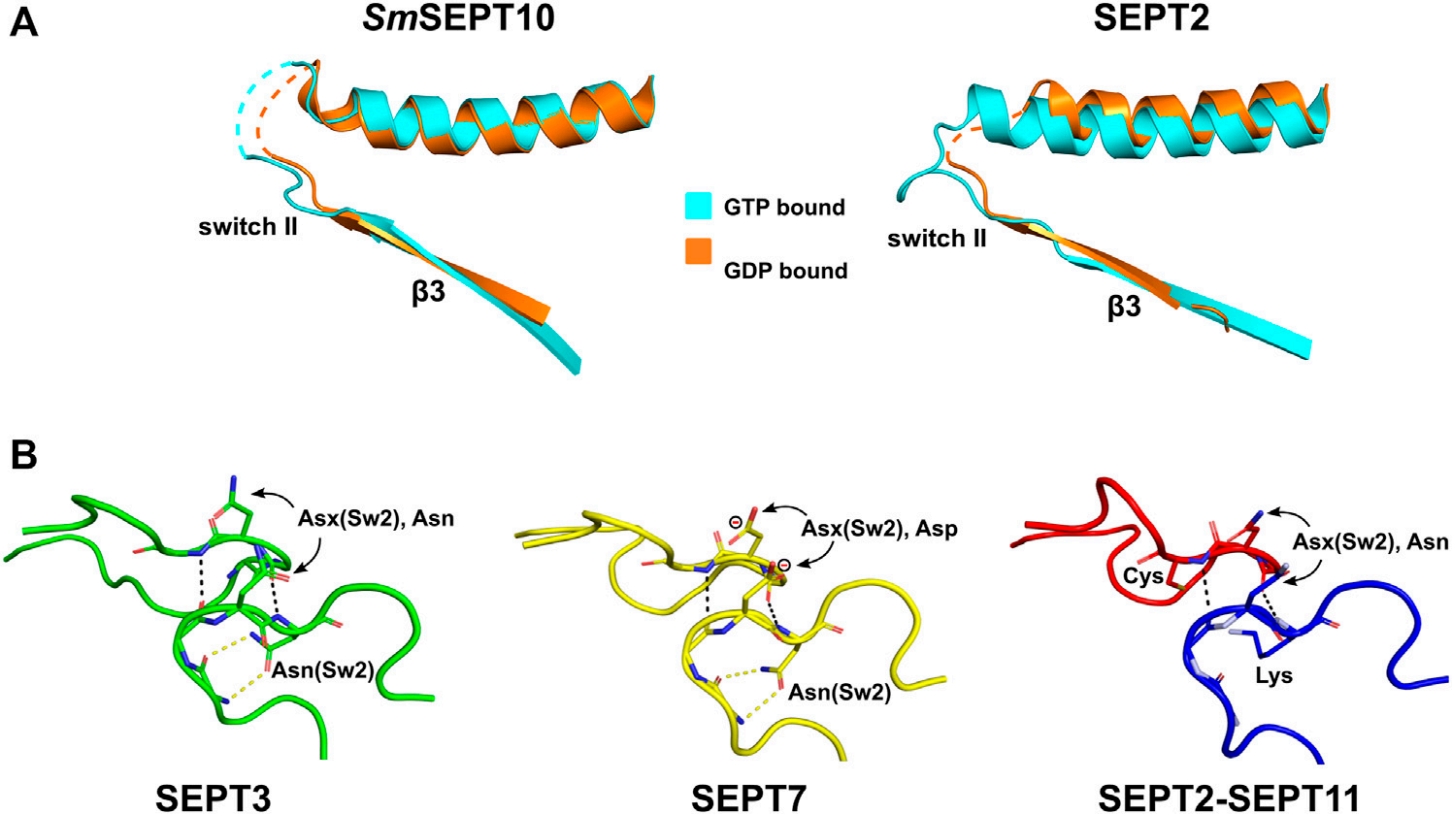
Switch II diversity at the G-interface. **(A)** β-slippage of the β3-strand in *Sm*SEPT10 and SEPT2. After GTP hydrolysis, the β3-strand slides in the direction of the G-interface, which prevents the formation of the correct contacts between the two copies of switch II (the β-bridge). PDB:4KVA (left; GTP, cyan), PDB:4KV9 (left; GDP, orange), PDB:3FTQ (right; GMPPNP, cyan), PDB:2QNR (right; GDP, orange). **(B)** The β-bridge (characterized by the hydrogen bonds shown as black dashed lines) in homotypic and heterotypic G-interfaces. Asx(Sw2) residues, conserved in septins, are paired across the interface. In SEPT7, this position is an aspartic acid (formal charge displayed). An asparagine residue, Asn(Sw2), conserved in the SEPT3 and SEPT7 groups, forms hydrogen bonds (yellow dashed lines) with the main chain, helping to stabilize the β-bridge. A lysine substitutes this aspargine in the SEPT6 group and cysteines appear in this position in SEPT1 and SEPT2, suggesting a new mechanism to stabilize the β-bridge in these cases. SEPT3, PDB:4Z54; SEPT7, PDB: 6N0B; SEPT2-11, PDB:6UPQ.

By comparing the GTP- and GDP-bound forms of the *Schistosoma* septin, *Sm*SEPT10, a mechanism, controlled by nucleotide hydrolysis, was described for β-strand slippage (Zeraik et al., 2014). When bound to GTP, the β3-strand is in the “non-slipped” state and switch II is partially ordered (forming the type II β-turns described in Section 5.1.2). However, in the GDP-bound state, β3 is slipped towards the G-interface by two residues causing a dramatic rearrangement of the hydrogen bonding within the sheet. As a consequence of slippage, switch II becomes disordered and the G-interface is partially destroyed (Figure 8A). This phenomenon was also described for SEPT2 (Valadares et al., 2017) and, in both septins, the homotypic G-interfaces are *promiscuous* (i.e., not predicted by the canonical model shown in Figure 1B). β-strand slippage was initially proposed as a mechanism for transmitting information from the G-interface to the NC-interface, forcing the α0 helix to change conformation (see Section 9 and Section 10.1) (Valadares et al., 2017).

The accumulation of many high-resolution structures for known physiological G-interfaces, including SEPT2-SEPT6/8/11, SEPT7-SEPT3_T282Y_ and SEPT7 alone (Brognara et al., 2019; Rosa et al., 2020), has forced this idea to be revised. In all these cases (as well as the homotypic interfaces formed by SEPT3-group members) the two switches are very well ordered (Supplementary Figure S3), forming a conserved wide-type β-bridge (Figure 4, Figure 8B) and the β3-strand remains unslipped. The available data suggests that the presence of the β-bridge is a consistent and necessary feature of a physiological G-interface and that slippage only occurs at *promiscuous* interfaces. Indeed, it has been suggested that slippage may be the result of “negative design” during evolution to disfavour *promiscuous* interfaces from forming *in vivo* (Brognara et al., 2019). In this sense, it is interesting that the SEPT3 group also presents a well ordered β-bridge and may be indicative of alternative subunit arrangements including homopolymers (Nakos et al., 2019b). On the other hand, the absence of β-strand slippage in septins of the SEPT3 group may instead be an artefact related to the presence of Mg^2+^ in the structure bound to GDP, which may aid in holding the strand in the non-slipped position (Castro et al., 2020).

The intermolecular β-bridge is stabilized by main chain hydrogen bonds, which place asparagine or aspartic acid residues (Asx(Sw2)) paired across the interface (Figure 8B). The main chain torsion angles observed for Asx(Sw2) are unusual and rarely adopted by other amino acids (Hovmöller et al., 2002), which suggests the β-bridge to be a unique structural motif in septins, differentiating them from other small GTPases. β-turns before and after the β-bridge aid in its correct orientation (Brognara et al., 2019; Rosa et al., 2020). SEPT3 and SEPT7 have an asparagine (Asn(Sw2)) immediately after Asx(Sw2), whose side chain forms hydrogen bonds in the homo- (SEPT3-3, SEPT7-7) and heterodimers (SEPT3-7), further stabilizing the structure (Figure 8B). Heterodimers of SEPT2 with SEPT6/8/11, on the other hand, present a cysteine (partially conserved for the SEPT2 group) or a lysine (conserved in the SEPT6 group) at this position (Figure 8B). This consequently eliminates the interactions made by the asparagines and suggests an alternative mechanism for stabilizing the structure in these cases. Rosa et al. (2020) have speculated that the side chains of the lysine and the cysteine, which face one another under the β-bridge, could potentially form a rare Lys-Cys covalent bond described for the first time only recently (Ruszkowski and Dauter, 2016; Wang, 2019; Wensien et al., 2021). Further investigation of such a bond forming *in vivo* and its potential relevance in stabilizing septin core particles is clearly necessary. Particularly interesting is the fact that the cysteine residue is not conserved in all human SEPT2-group members (only in SEPT2 itself and SEPT1) and this may provide a means to fine-tune inter-subunit affinities even within the paradigm imposed by Kinoshita’s rule.

## 7 The N-Terminal Domains

### 7.1 The Septin N-Terminal Domain and its Modular Features

The septin N-terminal domain is the least studied in terms of structure and the most variable region amongst all septins. This domain contains a structured α-helix (α0, preceding the G-domain), which often contains a polybasic basic region (PB1) believed to be crucial for membrane interaction (Zhang et al., 1999). However, the greater part of the septin N-domain, upstream to this helix, is intrinsically unstructured (Garcia et al., 2006). Some septins with long N-terminal prolongations (such as human SEPT4 and SEPT9) can be expressed as numerous alternatively spliced isoforms, giving rise to multiple possibilities and functional variation in the different tissues where they are present. Amongst mammalian septins, for example, SEPT9 possesses more than 30 different isoforms (Connolly et al., 2014; Zuvanov et al., 2019), and many functions have been shown to be isoform-specific (Estey et al., 2010; Connolly et al., 2011; Kim et al., 2011).

**FIGURE 9:**
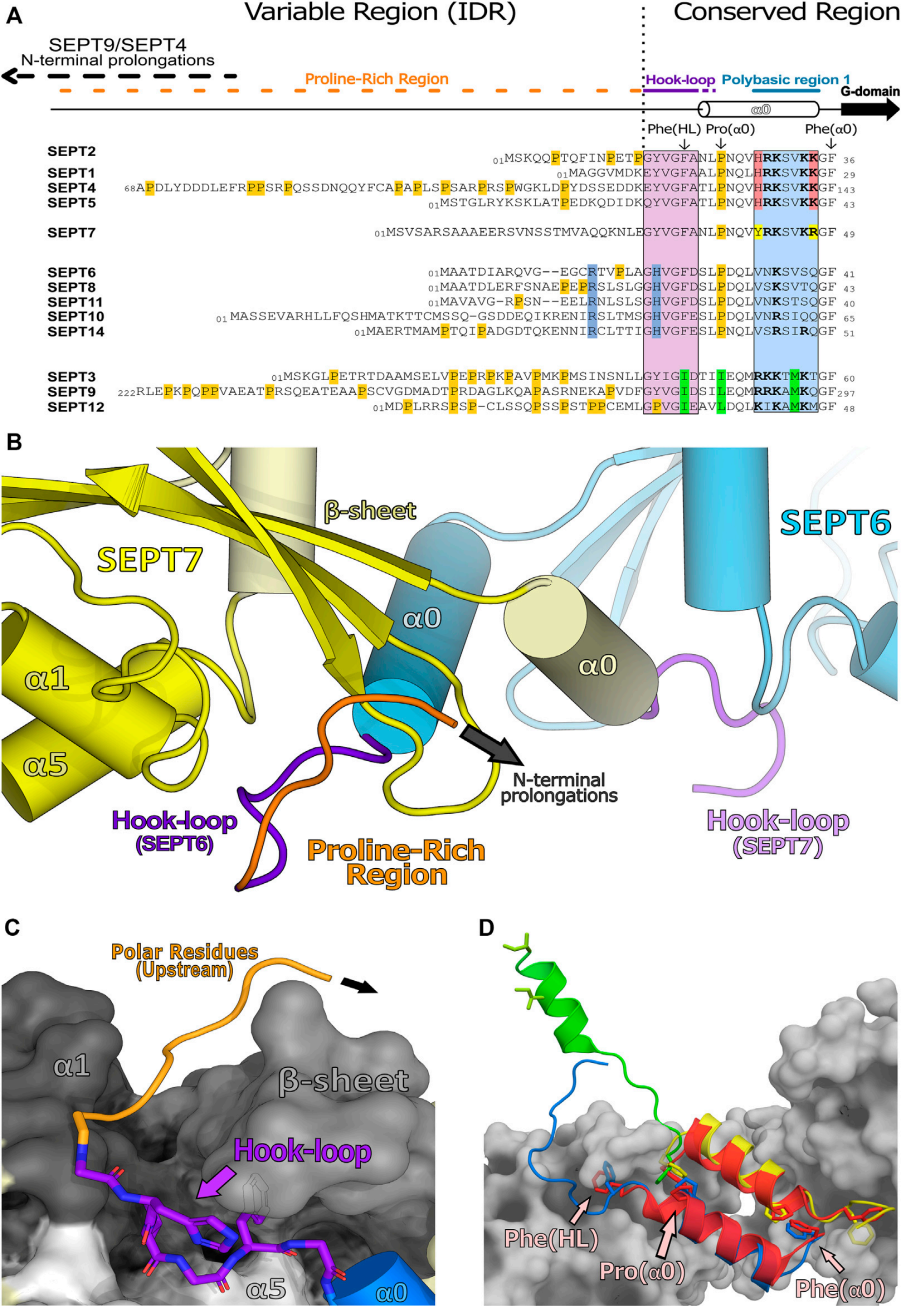
Human septin N-domains and their structured regions. **(A)** Alignment of all human N-terminal sequences showing the unstructured region (variable region or intrinsically disordered region, IDR) and the structured region (conserved region). This alignment also highlights the conserved residues within a septin group (also known as *characteristic* residues, in their respective colors) as well as proline residues (in orange). **(B)** Domain-swapped “hook-loop” highlighting the unstructured proline region (orange) and the hook-loop (purple) (PDB:7M6J). **(C)** Elements of SEPT7 (in greyscale) which accommodate the hook-loop of SEPT6 into a cleft. Residues with small side chains from the “hook-loop” are important for these interactions. **(D)** Helix α0 and its orientations within available septin structures (SEPT3 in green, PDB:4Z54; SEPT6 in blue-SEPT7 in yellow, PDB:7M6J; SEPT2 in red, PDB:2QA5). Also highlighted, as sticks, are residues which are essential for the NC-interface and/or the conformation of α0 (Phe(HL), Phe(α0) and Pro(α0); indicated in only one chain for clarity). These phenylalanines act as anchors and are conserved in SEPT2, SEPT6 and SEPT7 groups but are mutated to isoleucines in the SEPT3 group which may be related to additional α0 mobility in the latter.

A description of the N-domain can be made with reference to the characteristics of the conserved regions observed in mammalian septin sequences and highlighted in the alignment given in Figure 9A. Conveniently, this can be divided into two modules. Firstly, the unstructured (variable) region (IDR), including the prolongations observed for some SEPT4 and SEPT9 isoforms, whose long N-termini may have analogues in other species (Cdc3 in yeast or Pnut in *Drosophila*), along with some modulatory motifs within the downstream proline-rich region. Secondly, the structured (conserved) region consists of two components: 1) a domain-swapped loop (the “hook-loop”) important for NC-interface stabilization and 2) the α0 helix including PB1, the most conserved, structured and functionally characterized region of the N-domain.

### 7.2 The Extended SEPT9/SEPT4 N-Domains: Specificity for Protein-Protein Interactions

The N-domains observed in the longest isoforms of SEPT9 and SEPT4 possess specific motifs attributed to interacting with cytoskeletal proteins such as actin and microtubules (Bai et al., 2013; Smith et al., 2015; Verdier-Pinard et al., 2017). In particular, attempts have been made to divide the long domain found in SEPT9 into two distinct regions based on amino acid content. The first half is a basic domain containing a cytoskeletal binding region (CBR) involved in cytoskeletal protein recognition. The second half is more acidic and includes a proline-rich motif together with the structured (conserved) region (Supplementary Figure S4). The prolongations observed in these septins have also been shown to directly mediate interactions with proteins associated with other functions, such as vesicle trafficing (dynactin) (Kesisova et al., 2021) and signalling pathways (CIF15, SA-RhoGEF) (Nagata and Inagaki, 2005; Diesenberg et al., 2015). For a more detailed review on interactions involving the N-domain see Spiliotis and Nakos (2021).

### 7.3 Proline-Rich Motifs: Tuning Interactions and Functions

The proline-rich region, which follows the basic CBR, has also been credited with binding to different protein partners, thereby modulating protein interactions and functions. Some septin isoforms lacking the CBR present enhanced affinity towards signaling factors containing SH3 domains known to recognize proline-rich motifs. Many modulatory motifs and PTM sites have also been described within this region (such as acetylation, phosphorylation and SUMOylation motifs) (Van Damme et al., 2012; Zhou et al., 2013; Ribet et al., 2017). These modules could act as switches controlling and altering the effects of their flanking regions such as the CBR itself.

Shorter isoforms lacking the unstructured region were also shown to lose interaction specificities, and to increase binding to non-canonical paralogs and/or *promiscuous* partners, thereby, expanding their ordinary interactome (Devlin et al., 2021). It has been suggested that the presence of these proline-rich motifs (together with charged residues) (Supplementary Figure S4) might fine-tune NC-interface interactions and further restrict abnormal contacts and unusual filament assembly (Kim et al., 2012; Weems and McMurray, 2017; Jiao et al., 2020; Soroor et al., 2021).

### 7.4 Structured Regions Within the N-Domain

#### 7.4.1 The Domain-Swapped “Hook-Loop”

At the start of the structured region, there is a largely conserved motif (V/IGF/I), part of the “hook-loop” (HL, for short), connecting the unstructured proline-rich motif to the α0 helix. The hook-loop participates in domain-swapping, where it is buried in a groove formed by its NC-interface partner (Figure 9B). This cleft lies under the central β-sheet (β1, β2, β3) and is flanked by part of α1 and by one side of the C-terminal region of α5′ (Sirajuddin et al., 2007; Sirajuddin et al., 2009; Mendonça et al., 2021), stabilizing the interaction between NC partners (Figure 9C). Of particular note is the final hydrophobic residue of the motif, Phe(HL). In known structures, this phenylalanine, prior to α0, is buried in a hydrophobic pocket which aids in anchoring the helix within the NC-interface (Figure 9D; Sirajuddin et al., 2007). The preceding conserved glycine (Gly(HL)) may be necessary to give sufficient flexibility to allow the phenylalanine to uncouple from the pocket and release α0 from the interface when it closes (see Section 9 and Section 10.1). It seems likely that this motif emerged early in evolutionary history, since it is conserved to some extent even in paraseptins and probably existed in their common ancestor. However, in paraseptins, the motif is not used to accomplish domain swapping (Sun et al., 2002; Koenig et al., 2008).

#### 7.4.2 Alpha Zero and Polybasic Region 1

The α0 helix is sometimes considered to be part of the G-domain but here we include it in the N-domain, as both the helix itself and the G-domain lacking it, are able to fold independently. The α0 helix is an integral part of the NC-interface (see Section 9), where its structure has been best defined in the recent cryo-EM study of the SEPT2/6/7 hexamer (PDB:7M6J). The PB1 of α0, a stretch of seven residues, can be divided into two basic elements: proximal and distal. Four basic residues comprise PB1 in the SEPT2, SEPT3 and SEPT7 groups, but only one in the SEPT6 group, which therefore lacks a genuine polybasic region. In the SEPT2 and SEPT7 groups the four basic residues are divided equally between the proximal and distal regions and are distributed in an asymmetric fashion around the helix such that they are buried within the NC-interface, interacting with different components of the neighboring subunit (Mendonça et al., 2021). Furthermore, truncations within this region revealed its essential role not only for correct heterocomplex assembly and polymerization events but also for PIP selection during membrane association (Zhang et al., 1999; Casamayor and Snyder, 2003; Omrane et al., 2019; Taveneau et al., 2020).

The hook-loop, the α0 helix and the PB1 motif of the SEPT3 group present some unique properties when compared with their paralogues. The anchor Phe(HL) is replaced by an isoleucine, there is no proline in the first helical turn of α0 (Figure 9D) and the distribution of basic residues about the α0 helix is different from SEPT2 and SEPT7. These variations suggest that the N-terminal region of the SEPT3 group members may behave differently to other septins (see Section 9.3 and Section 10).

## 8 The C-Terminal Domains

The C-terminal domains of septins have long been associated with filament bundling. Models have been suggested in which these domains may form cross-bridges between neighboring filaments leading to higher-order complexes. It is often assumed that the coiled-coil regions within the domain have a major role in this process. However, it is only very recently that detailed structural information has become available for these domains and this is beginning to shed light on the organization of filaments and bundles.

### 8.1 Components Within the C-Terminal Domains

**FIGURE 10:**
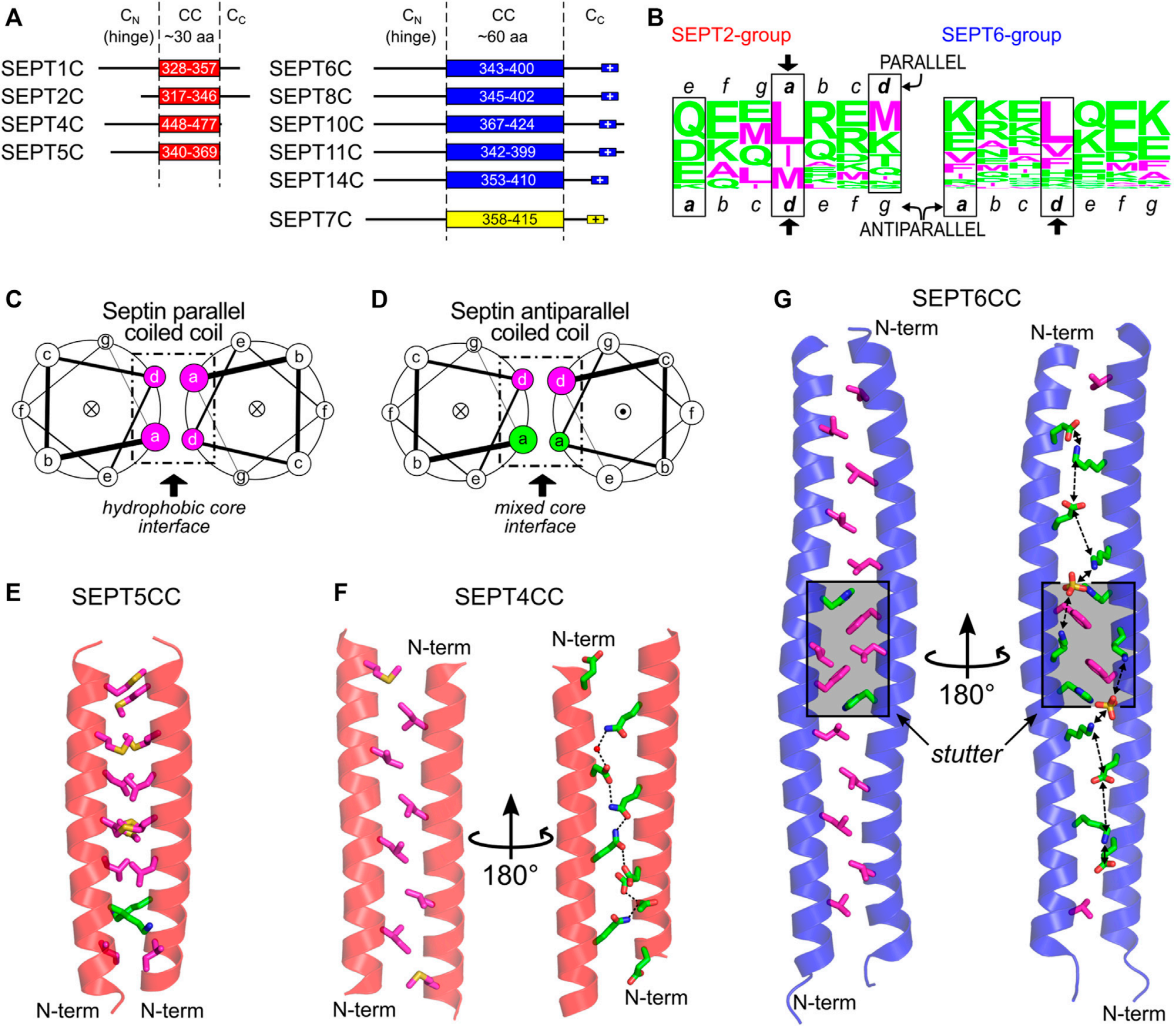
The C-domain and the septin coiled coils. **(A)** C-domain elements in SEPT2- (red), SEPT6- (blue) and SEPT7-group members (yellow) as reported by Leonardo et al. (Leonardo et al., 2021). The following Uniprot entries were used for residue numbering: SEPT1, Q8WYJ6; SEPT2, Q15019; SEPT4, O43236; SEPT5, Q99719; SEPT6, Q14141-2; SEPT7, Q16181; SEPT8, Q92599-2; SEPT11, Q9NVA2; SEPT14, Q6ZU15. **(B)** CC heptad motifs in members of human SEPT2 **(left)** and SEPT6 group **(right)** represented using a residue frequency sequence logo. Identified core positions are highlighted in boxes and register assignment is shown as seen in the X-ray structures. General helical wheel schemes for **(C)** parallel and **(D)** antiparallel human septin coiled coils, showing the different chemical composition of residues in the core (*a* and *d* positions). The dot (●) or cross (**×**) inside the circles represent the helix direction, outward- and inward-pointing, respectively. **(E)** The parallel septin coiled coil of SEPT5 from the SEPT2 group (PDB:6WCU) showing mainly hydrophobic *a* and *d* side chain residues as sticks. **(F)** The antiparallel septin coiled coil of SEPT4 from the SEPT2 group (PDB:6WB3) showing the two sides of the interface (*d*-side, left; *a*-side, right) and the chain of hydrogen bonds seen on the hydrophilic side. **(G)** Antiparallel septin coiled coil of SEPT6 (PDB:6WBP) showing the two sides of the interface (as in panel **(F)**, but including the aromatic residue of the stutter, in both views) and the chain of favorable salt bridges seen on the hydrophilic *a* side. The stutter region is highlighted in grey. Throughout the figure, magenta and green colors represent hydrophobic and hydrophilic residues, respectively.

Coiled-coil (CC) sequences are found in the C-terminal domain of most septins, with some exceptions (e.g., members of the human SEPT3 group and the yeast septin Cdc10). In humans, the length of the coiled coils varies among the different groups: in the SEPT2 group, it comprises around 30 residues, whilst in the SEPT6 and SEPT7 groups, it is approximately twice as long (Figure 10A; Leonardo et al., 2021). In yeast, there is a lack of direct structural information available and the output from prediction tools together with the information/assumptions adopted by different authors (Versele et al., 2004; Barth et al., 2008; Meseroll et al., 2013; Finnigan et al., 2015; Mela and Momany, 2019; Taveneau et al., 2020) means that it is unclear whether yeast septins share similar coiled-coil lengths or not (Supplementary Figure S5).

Apart from the coiled coil itself, there are potentially two flanking regions in the C-domain: C_N_, the region between the final helix of the G-domain (α6) and the coiled coil, and C_C_, the region subsequent to the coiled coil (Figure 10A). The C_N_ region is highly variable and believed to be quite flexible. It appears to act as a hinge, allowing the coiled coil to move with respect to the G-domain (Sirajuddin et al., 2007; Mendonça et al., 2021). This region in Shs1 (more specifically residues 350–445, Supplementary Figure S5) has been implicated in stabilizing octamers (Taveneau et al., 2020). Similarly, the region flanking the C-terminus of the coiled-coil (C_C_) is believed to be mostly unstructured. In the SEPT6 and SEPT7 groups, at the very end of the C-terminal domain, a polybasic sequence is present (K/RK/RDKxK/RKN/K and EKNKKKGK, respectively). This motif, together with other polybasic domains in septins (PB1 and PB2), could assist in membrane interaction. Although still under debate, some studies indicate that specific C-domains (or parts of them) do indeed have a role in membrane association (Zeraik et al., 2016; Cannon et al., 2019; Jiao et al., 2020; Woods et al., 2021).

### 8.2 The Coiled-Coil Motif in General

Coiled coils are present in a wide variety of proteins and can be described as super-helical assemblies of two or more α-helices coiled together (Lupas and Bassler, 2017; Woolfson, 2017). One of the main roles of coiled coils is to promote protein oligomerization. The hallmark of dimeric coiled-coil sequences is the presence of heptads, repetitions of seven amino acid residues dubbed *a*-*b*-*c*-*d*-*e*-*f*-*g*. Positions *a* and *d* are occupied mostly by hydrophobic residues, frequently leucine or isoleucine. These two positions form the hydrophobic core at the interface of the coiled coil and they interact with neighboring residues by “knobs-into-holes” contacts (Crick, 1953). In parallel dimeric coiled coils, the core interactions are *a*-*a* and *d*-*d*, creating mixed strips including both *a* and *d* side chains on both sides of the coiled-coil interface (Figure 10C and Supplementary Figure S6A). In antiparallel dimeric coiled coils, however, the contacts are *a*-*d* (i.e., an *a* residue from one helix paired with a *d* residue from the other). This places all side chains from the *a* residues on one side of the coiled-coil interface (and therefore the side chains from *d* residues on the other) (Figure 10D and Supplementary Figure S6B).

Other positions (*b*, *c*, *e*, *f*, *g*) are more exposed and are usually occupied by hydrophilic residues. Since two turns of a standard helix do not exactly match the heptad length, the helices pack into a left-handed super-coiled structure. However, insertion of non-canonical repeats with lengths other than seven may modify the packing angle between the helices (Brown et al., 1996; Gruber and Lupas, 2003). An insertion of four residues (called a stutter), for example, creates a block of 11 residues (7 + 4) and leads to the unwinding of the left-handed supercoil (Brown et al., 1996; Gruber and Lupas, 2003).

### 8.3 Coiled Coils in Septins

Not visible in the structures of human septin oligomers due to the flexibility of C_N_ (Sirajuddin et al., 2007; Mendonça et al., 2021), the C-domains are expected to participate in two different types of coiled coil along the filament: the SEPT2 homodimer and the SEPT6-SEPT7 heterodimer (in yeast, the Cdc11 homodimer and the Cdc12-Cdc3 heterodimer, Figure 1B), both at NC-interfaces. Given the directions of the final helices (α6) of the respective G-domains, and how they project perpendicular to the main filament axis, septin coiled coils were inferred to be parallel. FRET experiments show that the C-domains of SEPT6 and SEPT7 do indeed form heterodimeric parallel coiled coils (Low and Macara, 2006), even though they are capable of also assembling into homodimers (Almeida Marques et al., 2012; Sala et al., 2016). Circular dichroism data showed that the heterodimer is more stable than the homodimers (Almeida Marques et al., 2012; Sala et al., 2016), presumably due to unfavorable like-charge repulsion at *a* positions in the latter (Sala et al., 2016). No detailed structural evidence is currently available for the heterodimeric coiled coil, but it is expected that it would aid in guiding the correct assembly of the core particles (Almeida Marques et al., 2012; Meseroll et al., 2013; Sala et al., 2016). Concerning the C-terminal domain of SEPT2, it has been shown that its cleavage by Zika virus NS2B-NS3 protease is associated with mitotic defects in neural progenitor cells (Li et al., 2019), emphasizing the importance of the domain presumably in correct filament assembly.

Recently, the first structures of septin coiled coils were solved by X-ray diffraction. These were five homodimeric coiled coils of human SEPT1, SEPT4, SEPT5 (from the SEPT2 group), and SEPT6 and SEPT8 (from the SEPT6 group) (Leonardo et al., 2021). In the SEPT2-group structures, while SEPT5CC is a conventional parallel coiled coil with hydrophobic residues in *a* and *d* (Figures 10C,E), SEPT1CC and SEPT4CC (Figure 10F) are antiparallel (Figure 10D) and use a different contact interface which only partially overlaps with that observed for SEPT5CC. It has been suggested that this implies that the sequences are orientationally ambiguous (Leonardo et al., 2021). The residues which are common to the interface in both arrangements are shown by an arrow in Figure 10B and a similar pattern appears to be present in yeast septins. Additionally, the residues in *a*, which occupy *e* positions in the parallel form, are all hydrophilic (Figure 10B, note that the sequence register must be altered in order to preserve the standard definitions for the heptad positions). These establish hydrophilic contacts down the *a-*side of the interface, forming a chain of hydrogen bonds which interleaves acidic residues with glutamines thereby avoiding like-charge repulsion (Figure 10F) (Leonardo et al., 2021).

The two coiled-coil structures of the SEPT6 group (SEPT6, Figure 10G, and SEPT8) are also antiparallel. Both structures are very similar and, essentially, the same region forms the coiled coil, which confirms the intrinsic disorder of the flanking C_N_ and C_C_ regions since, for the SEPT8 construct, the entire C-domain was crystallized (Leonardo et al., 2021). Here, *a* positions are populated by lysines and glutamates. Since these residues form a strip down the *a-*side of the interface, this results in a chain of potential inter-helical salt bridges which would stabilize the dimer (Figure 10G). One particularity of these two structures (compared to the antiparallel structures of SEPT1CC and SEPT4CC) is the presence of a conserved stutter in the sequences of all SEPT6-group members (FE/DxL↓KxxH/Q, where the arrow indicates the break in the heptad register). The stutter decreases the supercoiling, leading to a structure in which the helices effectively lie side-by-side. This lack of supercoiling effectively maintains the side chains of equivalent register positions (*a* or *d*) on the same side of the interface along the entire length of the coiled coil (Figure 10G).

It has been suggested that the two orientations for the coiled coils could be metastable structures (Leonardo et al., 2021) and modulated by the chemical microenvironment in which the coiled coil is embedded. NMR studies with coiled-coil peptides from the SEPT2 group in aqueous solution show that they have a tendency to be parallel with a conventional hydrophobic interface (Leonardo et al., 2021). On the other hand, when antiparallel, the septin coiled coil buries hydrophilic residues inside the interface. This would only be expected to happen in an environment of low dielectric constant where solvent has been largely excluded, similar to that found inside crystals.

### 8.4 Coiled Coils and Filament Pairing/Bundling

Although apparently important for the stabilization of the NC-interface in the parallel orientation, the C-domains are not required for polymerization (Sirajuddin et al., 2007; Szuba et al., 2021), indicative of an additional role. They have also been associated with the formation of paired filaments, gauzes and stacked filament structures (Bertin et al., 2008; Bertin et al., 2010; Jiao et al., 2020; Szuba et al., 2021). Two kinds of spacing between filaments have been reported *in vitro* both in yeast and in mammalian septins: tight (∼5 nm) (Bertin et al., 2010; Jiao et al., 2020; Leonardo et al., 2021; Szuba et al., 2021) and loose (15–20 nm) (Frazier et al., 1998; Versele et al., 2004; Bertin et al., 2008, 2010; Leonardo et al., 2021). The former resembles the lengths of the coiled coils of the SEPT2-group members (4–5 nm). The wider spacing is compatible with the coiled coils of the SEPT6-and SEPT7-group members (8–11 nm) when taking into account contributions from unstructured parts of the C-domain (C_N_ and C_C_) (Leonardo et al., 2021). The former has also been proposed to arise from lateral contacts between G-domains (Szuba et al., 2021). However, in yeast, deleting the C-domain of Cdc11 or Cdc3/Cdc12 eliminated tighly- and loosely-paired filaments, respectively, supporting the involvement of specific C-domains in maintaining each type of spacing (Bertin et al., 2010).

**FIGURE 11:**
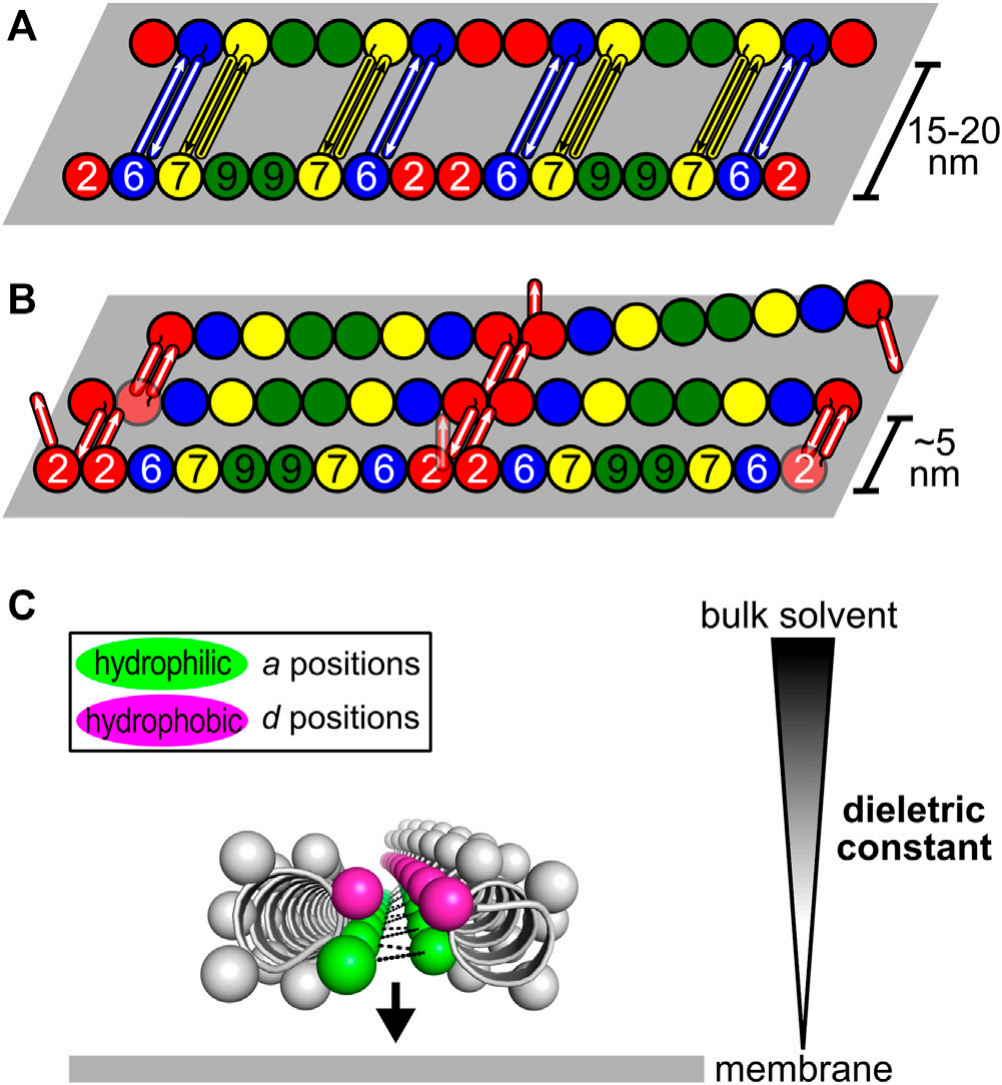
Model for how antiparallel coiled coils could mediate septin filament interconnections (Leonardo et al., 2021). The G- and the C-domains are represented as spheres and rods, respectively. **(A)** Loosely-paired filaments (or a “railroad-track”) separated by 15–20 nm are likely to be connected by antiparallel homodimers formed by the coiled coils of SEPT6 and SEPT7. **(B)** Tightly-paired sheets of filaments interconnected by antiparallel homodimers of SEPT2 coiled coils. **(C)** Solvent-protection of the hydrophilic side of the interface (*a* positions, green) in the antiparallel coiled coils. By facing the membrane, where the dielectric constant is lower than in bulk solvent, the formation of polar interactions is favored (black dashes).

A recent model attempts to relate the structural data on the predominantly antiparallel coiled coils to their potential role in mediating filament cross-bridging (Figures 11A,B; Leonardo et al., 2021). Previously, based on experiments with yeast septins, the formation of four-helix-bundles interconnecting filaments had been proposed (Bertin et al., 2008). However, evidence for tetrameric coiled coils has not been forthcoming (Almeida Marques et al., 2012; Leonardo et al., 2021). Additionally, the use of equimolar mixtures of the coiled-coil peptides from SEPT6 and SEPT7 in crystallization assays—expecting the crystallization of the heterodimer—yielded only antiparallel SEPT6 homodimers (Leonardo et al., 2021). The model shown in Figure 11 is a proposal for how both tight and loose spacings could arise by simple antiparallel pairings, which does not require the appearance of four-helix bundles. Although the details remain unknown, it is likely that the hydrophilic face (*a* positions) of these coiled coils would have to be solvent-protected to be stable, for instance by facing the membrane, where the dielectric constant is known to be lower (Figure 11C).

## 9 The NC-Interfaces

The polymerization of septin core particles in accordance with the canonical model generates either two or three chemically distinct NC-interfaces (Figure 1B). The SEPT6-SEPT7 NC-interface is common to both oligomers and has been most fully characterized in the cryo-EM structure of the SEPT2/6/7 complex (PDB:7M6J; Mendonça et al., 2021). The SEPT2-SEPT2 NC-interface is also common to both hexamers and octamers and is responsible for end-to-end polymerization. The SEPT3-SEPT3 NC-interface, unique to octamers, varies in terms of inter-subunit contacts and has been only partially described due to the lack of the α0 helix in most crystallized constructs.

### 9.1 SEPT6-SEPT7

**FIGURE 12:**
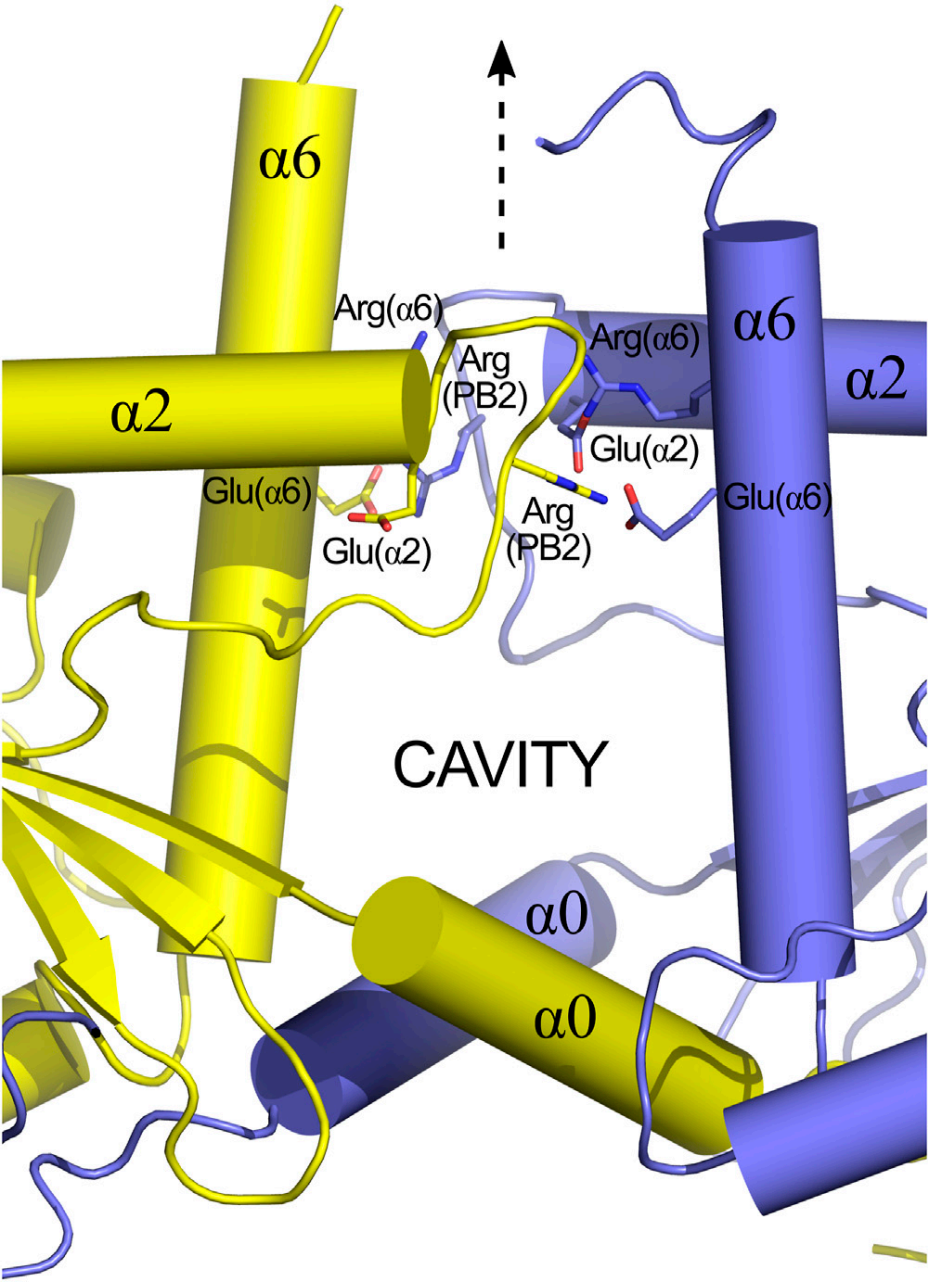
The NC-interface and its cavity (PDB:7M6J, chains D and E). SEPT6 and SEPT7 are coloured in blue and yellow, respectively. The pseudo symmetry diad is displayed (dashed arrow). The NC-interface has an upper part (indicated by residues around the C-terminus of helices α2) and the lower part (α0). In the upper part, four residues conserved in most septins are indicated on both subunits: Glu(α2), Arg(PB2), Glu(α6) and Arg(α6). The inter-subunit electrostatic interactions are made between Glu(α2) and Arg(PB2), Glu(α6) and Arg(PB2) and Arg(α6) with the helical dipole of helix α2.

Although the NC-interface between SEPT6-7 was originally reported in the crystal structure deposited in 2007 (PDB:2QAG; Sirajuddin et al., 2007), the low resolution of the data at the time precluded its full description. The cryo-EM structure (PDB:7M6J; Mendonça et al., 2021), taken together with high-resolution crystal structures of its components, reveals that the NC-interface can be divided into two regions: the upper part, where salt bridges are formed by residues from the α6 helix and the loop following α2, and the lower part, formed mainly by contacts made by helix α0. The two regions are connected by one face of helix α6 (Figure 12). The upper part of the interface has been extensively described previously (Valadares et al., 2017; Castro et al., 2020). It involves inter-subunit electrostatic interactions made between, Glu(α2) and Arg(PB2), Glu(α6) and Arg(PB2) and Arg(α6) with the helical dipole of helix α2, according to our simplified nomenclature (Figure 12; Sirajuddin et al., 2007; Macedo et al., 2013; Valadares et al., 2017).

Helix α0 of SEPT7 contains a genuine polybasic region (PB1), composed of seven residues, including four positive charges. The same region in SEPT6 contains only one (Figure 9A). In SEPT7, PB1 can conveniently be divided into a proximal region and a distal region, each containing two basic residues. Both regions form contacts with SEPT6 across the interface; the proximal region with helices α5 and α6 (including the elbow which connects them) and the distal region with the PAR.

One feature revealed by the cryo-EM structure is a large cavity at the SEPT6-7 NC-interface (Figure 12), whose perimeter is defined by the upper and lower contact regions together with α6. The bottom of the cavity is limited by a platform formed by the two α0 helices, held in place by the interactions described above, together with a phenylalanine anchor (Phe(α0)) at the boundary between α0 and the G-domain. It is interesting to note that the α0 helix is therefore anchored by two well-conserved phenylalanines at either end; Phe(HL) from the hook-loop (Section 7.4.1) and Phe(α0) at the interface with the G-domain. The cavity has no known function but is necessary for the monomers to be able to slide with respect to one another, although it is still unknown if this shifting is a general phenomenon which applies to all NC-interfaces or if it is restricted to the SEPT3 group alone (see Section 9.3 and Section 10.3). Other possible roles for the cavity cannot be eliminated, for example, in lipid binding during membrane association.

### 9.2 SEPT2-SEPT2

The homotypic SEPT2-SEPT2 NC-interface was partially described by the 3.4 Å structure of SEPT2 lacking the C-domain, reported in 2007 (PDB:2QA5; Sirajuddin et al., 2007). It is the main determinant of septin filament polymerization and its exposure at the ends of the oligomers permits the formation of filaments including a mixture of octameric and hexameric core particles. In this crystal structure, the upper part of the interface is similar to that described above. Indeed, the network of salt bridges involving charged residues from the α6 helix and the loop following α2 is a general feature of homologues in general, suggesting this to be a constant feature of all NC-interfaces.

In order to better understand the totality of the interface, we generated a SEPT2α0 model based on the highest resolution structure available (PDB:6UPQ) to which the α0 helix from PDB:2QA5 had been grafted. The SEPT2α0 NC-interface is in the open conformation in this model with α0 buried, as described for the case of SEPT6-7.

Like SEPT7, SEPT2 possesses a genuine PB1 with four positively charged residues divided into proximal and distal parts. Based on these observations, it has been suggested that similar interactions to those described for SEPT6-7 would be expected to participate across the interface (Mendonça et al., 2021). It appears clear from the model that interactions between PB1 and the PAR are to be anticipated. However, the SEPT2 NC-interface is now known to be the weakest link along the filament (at least in terms of its susceptibility to salt concentration) and molecular simulations appear to justify its fragility (Mendonça et al., 2019). In contrast, in terms of contact area and estimated free energy, the SEPT6-7 NC-interface (PDB: 7M6J) is estimated by PISA (Krissinel and Henrick, 2007) to be more stable than the SEPT2-2 NC-interface (2,149.8 Å^2^ and -16.6 kcal/mol, respectively, compared to 1,636.3 Å^2^ and −6.5 kcal/mol). Taken together, it is therefore possible to rationalize the rupture of filaments preferentially at homotypic SEPT2 interfaces at high ionic strength.

### 9.3 SEPT3-SEPT3

In all crystal structures presenting an NC-interface, the same canonical “open” conformation is observed, except for the SEPT3 group (Sirajuddin et al., 2007; Macedo et al., 2013; Castro et al., 2020). In these, the homotypic NC-interface is physiological and occupies the center of the octamer (Mendonça et al., 2019; Soroor et al., 2021). Its plasticity results in at least three different conformations: open, closed, and shifted (Macedo et al., 2013; Castro et al., 2020). The crystal structures of the SEPT3 G-domain (PDB:4Z51 and PDB:4Z54) complexed to either nucleotide are found to be in the closed form. For the G-domain of SEPT12 (PDB:6MQ9 and PDB:6MQK), both types of interface (open and closed) are found within the same filament, independent of the nucleotide, and the closed interface presents a displacement that breaks the twofold symmetry (the “shifted” conformation). In the case of SEPT9 (PDB:5CYP and PDB:5CYO), the conformation depends on the bound nucleotide, being open when bound to GDP and closed when bound to GTPγS (Macedo et al., 2013; Castro et al., 2020).

Since the closed conformation has thus far only been observed for SEPT3/9/12, it is tempting to believe that this is a unique property of the group. As mentioned above (Section 7.4), the N-terminal region in these septins presents some unique properties, including the charge distribution along α0 and the absence of one of the anchoring phenylalanines (Phe(HL)). These features could facilitate its exit from the cavity, allowing for the closure of the NC-interface, which would otherwise be impossible due to steric hindrance.

## 10 Domain Movements Within a Filament and Insights into Membrane Interaction

### 10.1 Squeezing of the Central NC-Interface

The canonical open conformation of the NC-interface is characterized by a ∼20 Å separation between the two α6 helices, which is reduced to approximately 12 Å in the SEPT3 group when the interface closes. This conformational change results from a rearrangement of the salt bridges at the upper part of the interface (Macedo et al., 2013; Castro et al., 2020). With the closure of the interface, the PB2 region, which follow α2, wraps around α6 of its neighbor and approaches the polyacidic region (PAR). Specifically, Arg(PB2) dips down into the interface forming a new salt bridge with a glutamic acid from the PAR (Macedo et al., 2013; Castro et al., 2020). In this new conformation, Glu(α2) interacts directly with Arg(α6), which no longer interacts with the α2 helix dipole (Macedo et al., 2013; Castro et al., 2020). As a consequence, the α6 helices approach one another while the α2 helices move apart.

With the closure of the interface, the α0 helix is displaced out of the NC-interface, thereby gaining conformational freedom (Castro et al., 2020). This is not only suggested by the steric hindrance, which would result if α0 were to remain within the interface but has actually been observed experimentally in one of the crystal structures of SEPT3 (PDB:4Z54). Different to when hidden within the interface, where its positive charges are inward-pointing and occupied in stabilizing the interface itself, once liberated, PB1 would be free to interact with membranes (Zhang et al., 1999; Bertin et al., 2010).

**FIGURE 13:**
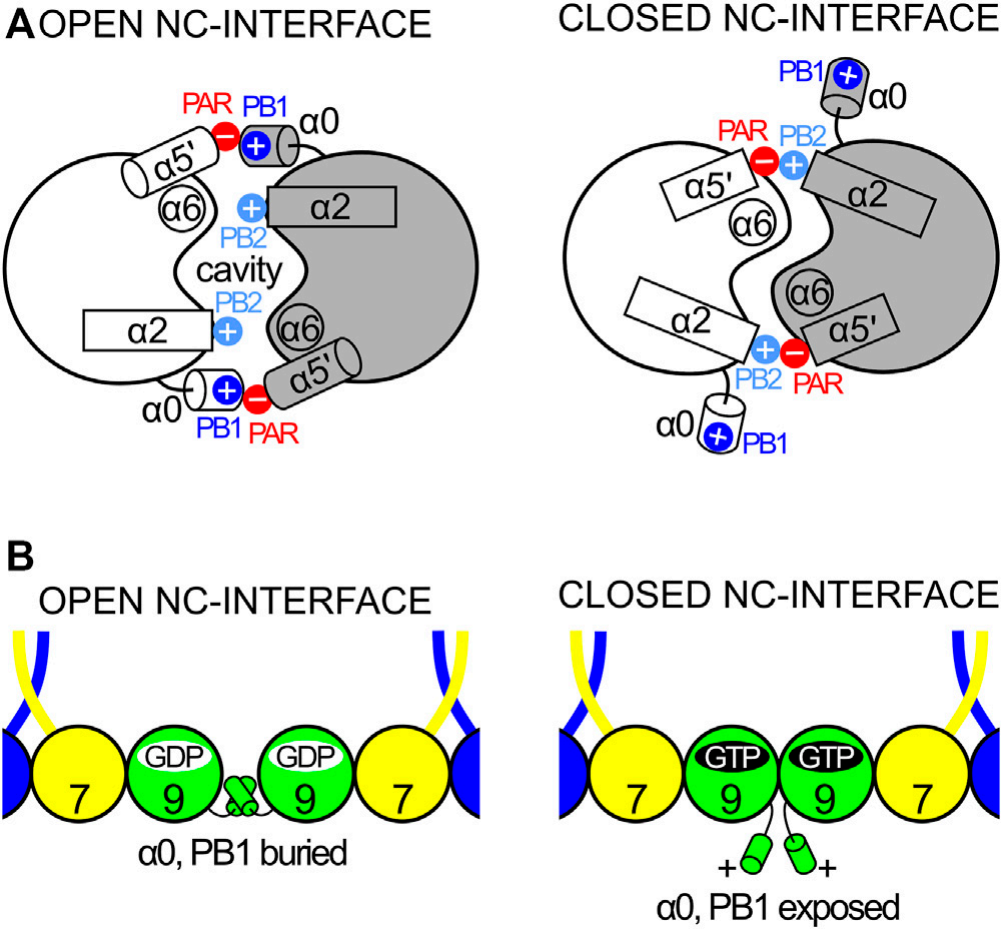
The dual role of the PAR, the exposure of PB1 and the concertina movement at the NC-interface. **(A)** When the interface is open, the PAR interacts with PB1 (left) but when closed with PB2 (right). **(B)** The closure of the interface results in exposing α0 and its associated PB1, releasing it from its buried (inactive) state in the NC-interface and enabling it for membrane association.

Figure 13 schematizes the differences between the open and closed states. Under this proposal, the PAR plays two important roles: 1) harboring α0 and its PB1 when buried in the open state and 2) stabilizing the closed conformation by interacting with PB2. As such, the open conformation appears incompatible with membrane association by PB1, but rather is necessary for its safe storage when this is not required (Castro et al., 2020). This mechanism is also compatible with a role for PB2 in membrane association on interface closure. As this occurs, and PB2 wraps around helix α6 of the neighboring subunit to interact with the PAR, it becomes more exposed on the filament side where it could act in concert with PB1 in membrane association. Indeed, its role as such has already been proposed (Omrane et al., 2019) and is compatible with the sense of bending of the hexamer observed by cryo-EM (PDB:7M6J) (see Section 10.3).

In all protein structures from the SEPT3 group, the α5′ helix is oriented differently from that observed for other septins, lying roughly parallel to the filament axis. This is due to the presence of *characteristic* residues such as *cis*Pro(α4-α5′/SEPT3), which result in the N-terminus of α5′ coming closer to α6 (Castro et al., 2020). This displaces the PAR upwards in a way which would favor interacting with PB2 in the closed conformation. The combined differences observed in the SEPT3 group, including those associated with α0, α5′ and the lack of a C-terminal coiled coil, may sum to give this NC-interface its apparently unique plasticity.

### 10.2 Communication Between Adjacent Interfaces and Information Transfer

The closed structure of the G-domain of SEPT9 (PDB:5CYP) was obtained by soaking a GDP-bound crystal (in the open conformation) with the GTP analogue, GTPγS, suggesting that the occurrence of nucleotide hydrolysis in the G-interface could result in conformational changes to the adjacent NC-interface. How might this occur now that β-strand slippage appears to have been eliminated as a potential conduit at physiological interfaces (see Section 6.3)? One possibility is helix α2, which runs from the switch II region of the G-interface at its N-terminus to the PB2 region of the NC-interface at its C-terminus. The septin specific sequence Sep2 (Pan et al., 2007) runs underneath this helix such that it is unable to pack against the central β-sheet (see Section 5.1.3). This unusual arrangement (Chothia et al., 1981) may free up α2 and allow it to move with respect to the rest of the structure, potentially as a rigid body or rod. As such, the conformational changes which occur to switch II on GTP hydrolysis could be more readily transmitted via α2 to the neighboring NC-interface. Although speculative, this mechanism would provide a functional role for the Sep2 motif, justifying its strict conservation in septins during evolution (Pan et al., 2007).

Nothing is known about the mechanism of information transfer itself. However, a specific aromatic residue of the switch II region, Aro(Sw2), appears to be a potential candidate. The SEPT3 septins bound to non-hydrolyzable GTP analogues currently represent the best model available for the pre-hydrolysis state. In all such structures, this aromatic residue lies such that the plane of the aromatic ring is parallel to the surface of helix α2. However, structures in the presence of GDP suggest that nucleotide hydrolysis perturbs the switch II region, causing Aro(Sw2) to assume an alternative conformation in which the ring lies approximately perpendicular to the helix surface (Supplementary Figure S7; Rosa et al., 2020). The change to the Aro(Sw2) rotamer appears to be directly coupled to a second aromatic residue, Phe(α3), on the inner surface of helix α3. This aromatic cluster rearrangement lifts α2 further from the underlying β-sheet, potentially giving it the necessary freedom to move and thus perturb the neighbouring NC-interface towards which it is slightly shifted (Supplementary Figure S7). The notion that this aromatic cluster could be essential for communication gains support from the fact that Phe(α3) is lacking in the catalytically inactive septins where, by definition, such a mechanism would be inoperative anyway. It is interesting to note that the information transfer may be due to a transitory perturbation to α2 rather than a switch between two well-defined states, an idea supported by the fact that the shifts described above are rather subtle. Clearly, more work is necessary on this point.

### 10.3 Transverse Modes of Filament Flexibility

**FIGURE 14:**
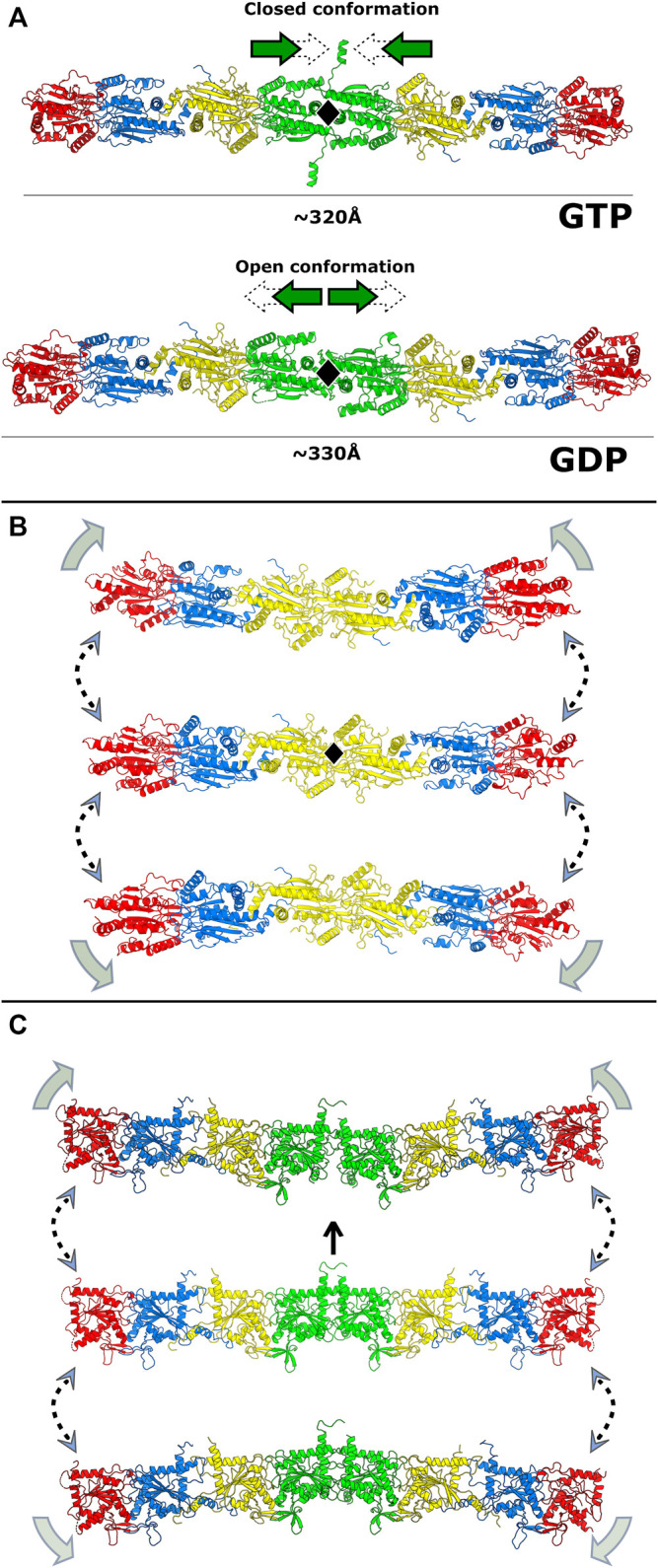
Intrinsic flexibility of the filament. **(A)** The proposed concertina movement of the octamer due to the opening and closing of the central SEPT3-group NC-interface. In this case, the twofold symmetry is preserved. **(B)** Flexion of the hexamer around the twofold axis between the two copies of SEPT7 at the center (yellow) present in the cryo-EM model (PDB:7M6J). The centre figure shows a linear particle which has a strict twofold axis perpendicular to the page (black lozenge). Above and below are shown bent particles. **(C)** Proposed flexion of the octamer, based on variations to the central NC-interface observed in crystal structures involving SEPT3-group members. In the central figure, the twofold axis is vertical (arrow). The upper complex is based on a central closed and shifted interface (PDB:6MQK) and the lower complex on the heterodimeric structure of SEPT7-SEPT3_T282Y_ (PDB:6UQQ).

The concertina movement due to opening and closing of the NC-interface of the SEPT3-group septins (Figure 14A) is not the only structural flexibility of the core particles for which there is experimental evidence. Although the flexibility of the hexamer had been noted previously (Sirajuddin et al., 2007), the study of Mendonça et al. (2021) was able to attribute this principally to movement at the central G-interface (SEPT7-SEPT7) (Figure 14B). The direction of greatest bending corresponded to flexion of the particle around the twofold axis which relates the two trimers (Figure 14B, center). Interestingly, this bending may be related to the recognition and/or interaction of the filaments with membranes. If the curvature of the oligomer were coincident with that of the membrane, it would lie laterally such that the main aperture to the cavity at the NC-interface would face the membrane. This raises intriguing possibilities, including, for example, that both PB1 and PB2 could interact simultaneously with negatively charged membrane lipids and that the cavity may play a role in this process. Obviously, the current scarcity of structural data only allows for speculation at this point and any attempt to draw a definitive conclusion would be premature.

Membrane recognition by septins can also be curvature-dependent, as has been shown for both yeast and animal septin complexes (Palander et al., 2017; Beber et al., 2019; Cannon et al., 2019; Woods et al., 2021). *In vitro* studies have shown that septin filaments preferentially bind to regions of curvature on the scale of microns (Bridges et al., 2016). Although the bending observed in the cryo-EM study only gives an indication of the direction of flexion and not its extent, nevertheless this would appear to be compatible with micron-scale recognition. Propagating the flexion observed at the central interface to all oligomers along a filament would easily produce ring-like structures with diameters compatible with the preferential curvatures already observed (Kinoshita et al., 2002; Tanaka-Takiguchi et al., 2009; Bridges et al., 2016; Beber et al., 2019).

Since the exposure of PB1 due to NC-interface closure is potentially a unique property of the SEPT3 group, this makes the conformational properties of the octameric particle of particular interest. At present, there are no experimental structures available for an octamer from any species. Nevertheless, the accumulation of partial structures of single septins and heterodimers, when taken together with that for the hexamer, mean that reliable models for the human octamer can be computationally generated. Figure 14C shows examples of how the variation observed at the central interface in the different crystal structures involving SEPT3-group members leads to full particles which present considerable structural diversity.

Clearly, structural studies of octameric particles with a view to determining the extent and direction of bending will be essential to understanding the physical properties of filaments. How these properties may depend on the ratio of hexamers to octamers is an intriguing question to be answered. More important still is how these relate to septin association with membranes and the cytoskeleton and thereby impact on septin function.

## 11 Future Directions

In a review article published in 2017 (Valadares et al., 2017) it was suggested that single-particle cryo-electron microscopy would inevitably play a significant role in the future of the structural biology of septins. With the recent publication of the first structure of a hexameric particle at 3.6 Å, it would seem that this is already coming true. Undoubtedly, in the near future, cryo-electron tomography and subtomogram averaging of *in situ* samples will be able to provide a more realistic view of intracellular septin localization, function and dynamics and it is exciting to look forward to the future, knowing that these technologies are both powerful and robust.

As this review is being written, free worldwide access to the AI structure predictor, AlphaFold2 (Jumper et al., 2021), is already a reality and it stands to revolutionize the way we think about structural biology and the information it carries about complex biological systems, septins included. Overnight, structures with accuracies which are likely to be close to experimental have been made available for all septins from humans, yeast, *Drosophila* and *C. elegans.* Whereas structural information has been largely restricted to human septins up until now, this will no longer be the case in the future. What is needed, more than ever, is the ability to interrogate these structures in order to glean relevant biological insight. The challenge will be to understand how septins associate, how they are regulated and modulated, how they respond to their microenvironment and how the dynamics of monomers, oligomers, filaments and bundles is associated with their interactions with membranes, the cytoskeleton and their other binding partners. Much progress has been made in understanding the structure-function relationships of septins over recent years, but there is still plenty to be done. Rather than further dissecting septin filaments into their component parts, it is now incumbent on those active in the field to integrate current and future information into a more complete picture of the complex biological systems in which septins participate.
