# Supplementary material for: The Structural Biology of Septins and Their Filaments: An Update
Source: Front Cell Dev Biol. 2021 Nov 19;9:765085. doi: 10.3389/fcell.2021.765085 (PMC8640212; doi:10.3389/fcell.2021.765085)
Supplement: Supplementary file 1 [file DataSheet1.pdf]

Supplementary Material

1. Supplementary Figures

(this page was intentionally left blank)

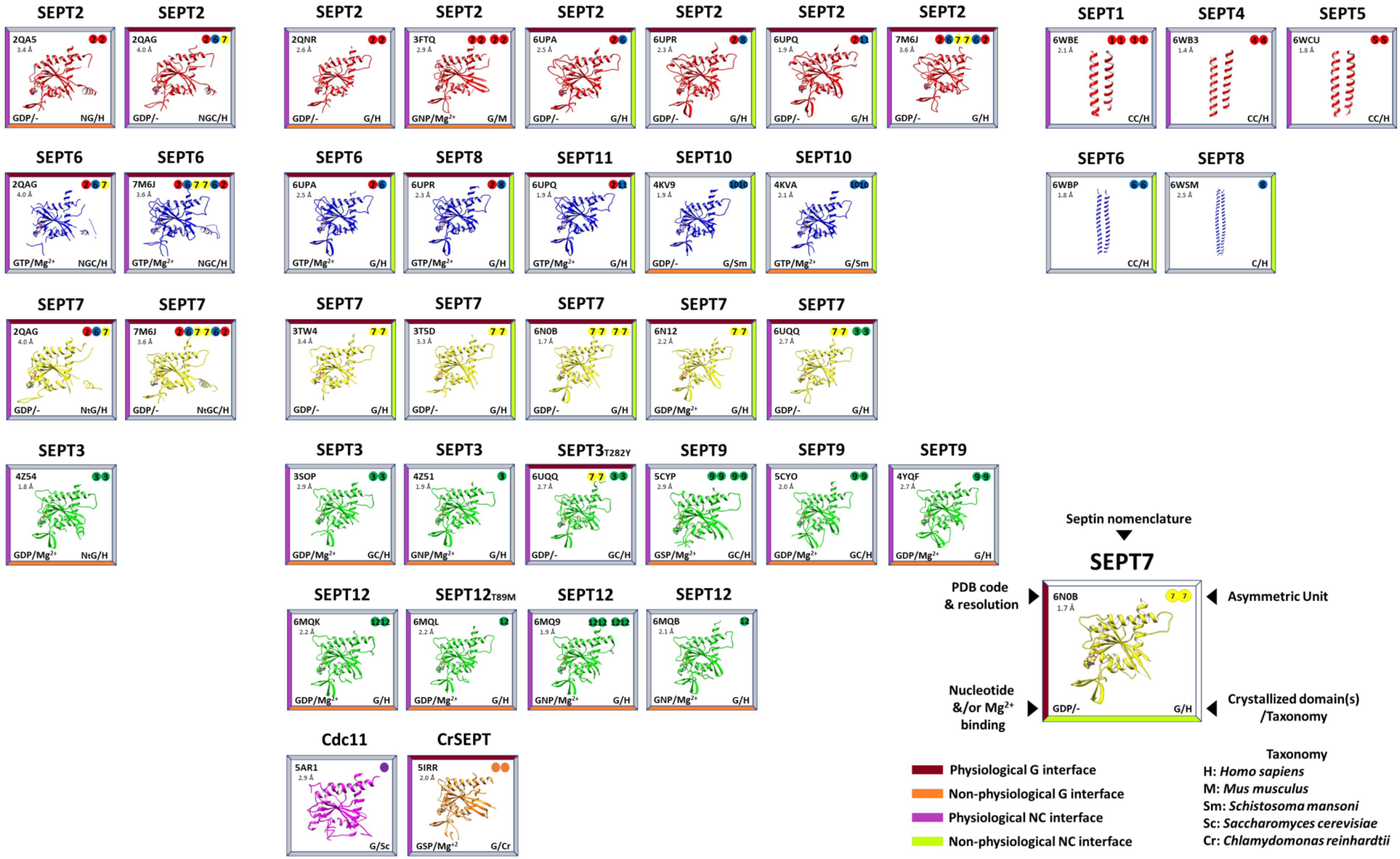
SEPT2
2QA5
3.4 Å
GDP/-
NG/H
SEPT2
2QAG
4.0 Å
GDP/-
NGC/H
SEPT2
2QNR
2.6 Å
GDP/-
G/H
SEPT2
3FTQ
2.9 Å
GNP/Mg2+
G/M
SEPT2
6UPA
2.5 Å
GDP/-
G/H
SEPT2
6UPR
2.3 Å
GDP/-
G/H
SEPT2
6UPQ
1.9 Å
GDP/-
G/H
SEPT2
7M6J
3.6 Å
GDP/-
G/H
SEPT1
6WBE
2.1 Å
CC/H
SEPT4
6WB3
1.4 Å
CC/H
SEPT5
6WCU
1.8 Å
CC/H
SEPT6
2QAG
4.0 Å
GTP/Mg2+
NGC/H
SEPT6
7M6J
3.6 Å
GTP/Mg2+
NGC/H
SEPT6
6UPA
2.5 Å
GTP/Mg2+
G/H
SEPT8
6UPR
2.3 Å
GTP/Mg2+
G/H
SEPT11
6UPQ
1.9 Å
GTP/Mg2+
G/H
SEPT10
4KV9
1.9 Å
GDP/-
G/Sm
SEPT10
4KVA
2.1 Å
GTP/Mg2+
G/Sm
SEPT6
6WBP
1.8 Å
CC/H
SEPT8
6WSM
2.5 Å
C/H
SEPT7
2QAG
4.0 Å
GDP/-
NtG/H
SEPT7
7M6J
3.6 Å
GDP/-
NtGC/H
SEPT7
3TW4
3.4 Å
GDP/-
G/H
SEPT7
3T5D
3.3 Å
GDP/-
G/H
SEPT7
6N0B
1.7 Å
GDP/-
G/H
SEPT7
6N12
2.2 Å
GDP/Mg2+
G/H
SEPT7
6UQQ
2.7 Å
GDP/-
G/H
SEPT3
4Z54
1.8 Å
GDP/Mg2+
NtG/H
SEPT3
3SOP
2.9 Å
GDP/Mg2+
GC/H
SEPT3
4Z51
1.9 Å
GNP/Mg2+
G/H
SEPT3T282Y
6UQQ
2.7 Å
GDP/-
GC/H
SEPT9
5CYP
2.9 Å
GSP/Mg2+
GC/H
SEPT9
5CYO
2.0 Å
GDP/Mg2+
GC/H
SEPT9
4YQF
2.7 Å
GDP/Mg2+
G/H
SEPT12
6MQK
2.2 Å
GDP/Mg2+
G/H
SEPT12T89M
6MQL
2.2 Å
GDP/Mg2+
G/H
SEPT12
6MQ9
1.9 Å
GNP/Mg2+
G/H
SEPT12
6MQB
2.1 Å
GNP/Mg2+
G/H
Cdc11
5AR1
2.9 Å
G/Sc
CrSEPT
5IRR
2.0 Å
GSP/Mg+2
G/Cr
Septin nomenclature
SEPT7
PDB code & resolution
6N0B
1.7 Å
Asymmetric Unit
Nucleotide &/or Mg2+ binding
GDP/-
G/H
Crystallized domain(s) /Taxonomy
Physiological G interface
Non-physiological G interface
Physiological NC interface
Non-physiological NC interface
Taxonomy
H: Homo sapiens
M: Mus musculus
Sm: Schistosoma mansoni
Sc: Saccharomyces cerevisiae
Cr: Chlamydomonas reinhardtii

Supplementary Figure 1. The “Septin Chart” including all septin structures published to date. The square sides which enclose each structure are coloured according to the presence of each interface: left side, physiological G-interface (burgundy); right side, physiological NC-interface (purple); upper side, non-physiological G-interface (orange); lower side, non-physiological NC-interface (lime); i.e., the indications of physiological interfaces are related to the pose in which the monomer is depicted (left, G-side; right, NC-side). Domain acronyms: N, N-terminal domain; Nt, truncated N-terminal domain; G, GTP-binding domain; C, C-terminal domain; CC, coiled coil. PDB:6MQ9 and PDB:5CYO should be preferred over PDB:6MQB and PDB:4YQF since they represent resolution-improved models.

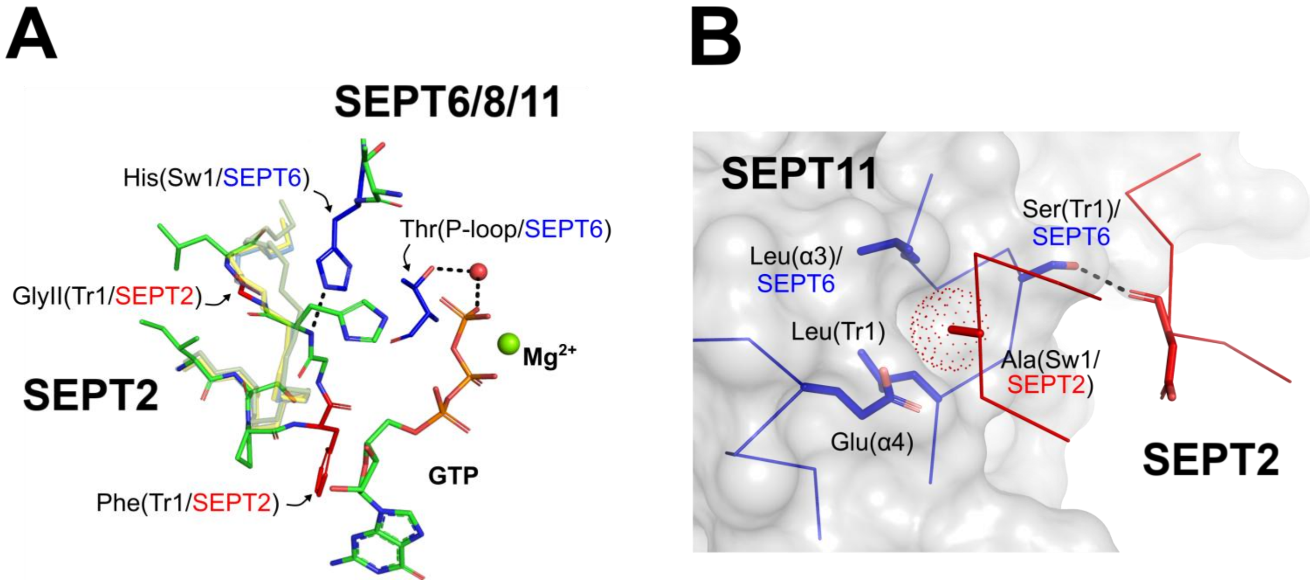

Supplementary Figure 2. Group-specificities within the G-interface formed by SEPT6-group members with SEPT2. *Characteristic* amino acids are coloured in blue (SEPT6 group) or red (SEPT2). (**A**) 3D structure showing how His(Sw1/SEPT6) interacts with the *trans*-loop 1 of SEPT2. The *characteristic* amino acids allow *trans*-loop 1 to adopt a different orientation from that seen in septins from other groups (transparent traces with SEPT3 in light green, SEPT7 yellow and SEPT11 in light blue). (**B**) The SEPT2 switch I region interacting with a cavity in SEPT11. The methyl of the *characteristic* amino acid Ala(Sw1/SEPT2) fits into the cavity formed by the residues shown (PDB:6UPQ).

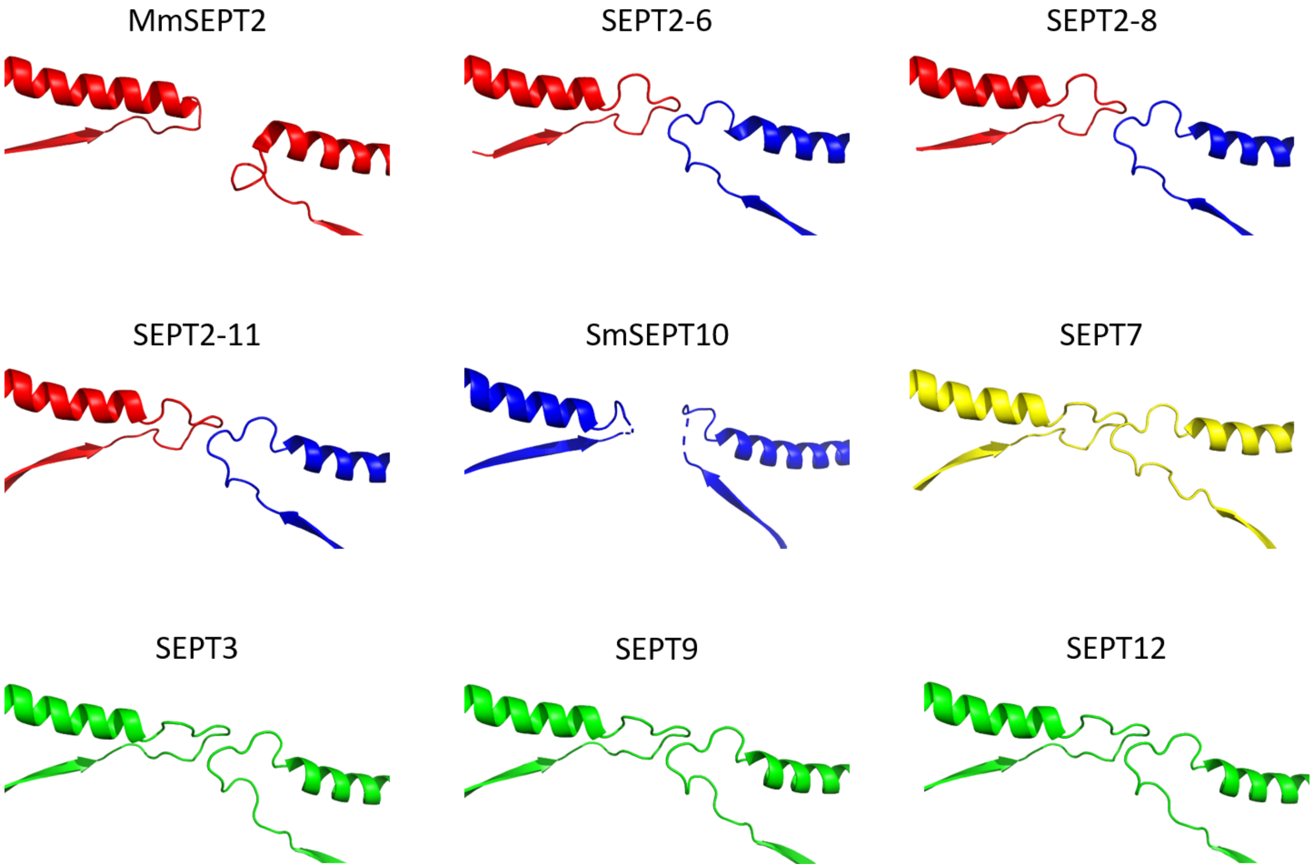

Supplementary Figure 3. Switch II organization in various septin structures. PDB IDs in reading order: PDB:3FTQ, PDB:6UPA, PDB:6UPR, PDB:6UPQ, PDB:4KV9, PDB:6N12, PDB:4Z54, PDB:5CYO and PDB:6MQK.

A

Alignment of N-terminal domains from Septin 9 Isoforms

Basic domain (Cytoskeletal Binding Regions - CBRs)

(Cytoskeletal Binding Region - CBR)

Long-isoform NTE Specificities

```
Q9UHD8-1|SEPT9 i1     1 -----------MKKSYSGGTRTSSGRLRRLGDSSGPALKRSFEVEEVETPNSTPPRRVQTPLLRATVASSTQKFQDLGVKNSEPSARHVDSLSQRSPKASLRRVELSGPKAAEPVSRRTELSIDISSKQVENAGAIGPSRFGLKRAEVLGHKT 142
Q9UHD8-5|SEPT9 i2     1 ------------------MSDPAVNAQLDGIISDFEALKRSFEVEEVETPNSTPPRRVQTPLLRATVASSTQKFQDLGVKNSEPSARHVDSLSQRSPKASLRRVELSGPKAAEPVSRRTELSIDISSKQVENAGAIGPSRFGLKRAEVLGHKT 135
Q9UHD8-2|SEPT9 i3     1 -----------------------------MERDRISALKRSFEVEEVETPNSTPPRRVQTPLLRATVASSTQKFQDLGVKNSEPSARHVDSLSQRSPKASLRRVELSGPKAAEPVSRRTELSIDISSKQVENAGAIGPSRFGLKRAEVLGHKT 124
Q9UHD8-3|SEPT9 i4     1 --------------------------------------------------------------------------------------------------------------------------------------------------------- 1
Q9UHD8-4|SEPT9 i5     1 --------------------------------------------------------------------------------------------------------------------------------------------------------- 1
```

Acidic Domain (Proline-Rich Region + Structured Region)

Proline Rich Region — Structured Region

```
Q9UHD8-1|SEPT9 i1   143 PEPAPRRTEITIVKPQESAHRRMEPPASKVPEVPTAPATDAAPKRVEIQMPKPAEAPTAPSPAQTLENSEPAPVSQLQSRLEPKPQPPVAEATPRSQEATEAAPSCVGDMADTPRDAGLKQAPASRNEKAPVDFGYVGIDSILEQMRRKAMKQ 295
Q9UHD8-5|SEPT9 i2   136 PEPAPRRTEITIVKPQESAHRRMEPPASKVPEVPTAPATDAAPKRVEIQMPKPAEAPTAPSPAQTLENSEPAPVSQLQSRLEPKPQPPVAEATPRSQEATEAAPSCVGDMADTPRDAGLKQAPASRNEKAPVDFGYVGIDSILEQMRRKAMKQ 288
Q9UHD8-2|SEPT9 i3   125 PEPAPRRTEITIVKPQESAHRRMEPPASKVPEVPTAPATDAAPKRVEIQMPKPAEAPTAPSPAQTLENSEPAPVSQLQSRLEPKPQPPVAEATPRSQEATEAAPSCVGDMADTPRDAGLKQAPASRNEKAPVDFGYVGIDSILEQMRRKAMKQ 277
Q9UHD8-3|SEPT9 i4     1 ----------------------MEPPASKVPEVPTAPATDAAPKRVEIQMPKPAEAPTAPSPAQTLENSEPAPVSQLQSRLEPKPQPPVAEATPRSQEATEAAPSCVGDMADTPRDAGLKQAPASRNEKAPVDFGYVGIDSILEQMRRKAMKQ 131
Q9UHD8-4|SEPT9 i5     1 -------------------------------------------------------------------------------------------------------------MADTPRDAGLKQAPASRNEKAPVDFGYVGIDSILEQMRRKAMKQ 44
```

B

Alignment of N-terminal domains from Septin 4 Isoforms

Long-isoform NTE specificities — Siah-1 Interacting Motif — Proline Rich Region — Structured Region

```
O43236-4|SEPT4             MRSSPALFSSRAAPQKPRKEGSQAAGLLVFSDSLEIKRFLEDTTDDGELSKFVKDFSGNASCHPPEAKTWASRPQVPEPRPQAPDLYDDDLEFRPPSRPQSSDNQQYFCAPAPLSPSARPRSPWGKLDPYDSSEDDKEYVGFATLPNQVHRKSVKK 156
O43236-1|SEPT4_i1          ---------------MDRSLGWQGNSVPEDRTEAGIKRFLEDTTDDGELSKFVKDFSGNASCHPPEAKTWASRPQVPEPRPQAPDLYDDDLEFRPPSRPQSSDNQQYFCAPAPLSPSARPRSPWGKLDPYDSSEDDKEYVGFATLPNQVHRKSVKK 141
O43236-3|SEPT4             -----------------------MPGFYSVMTDEEIKRFLEDTTDDGELSKFVKDFSGNASCHPPEAKTWASRPQVPEPRPQAPDLYDDDLEFRPPSRPQSSDNQQYFCAPAPLSPSARPRSPWGKLDPYDSSEDDKEYVGFATLPNQVHRKSVKK 133
O43236-2|SEPT4_i2(ARTS)----------------------------------MIKRFLEDTTDDGELSKFVKDFSGNASCHPPEAKTWASRPQVPEPRPQAPDLYDDDLEFRPPSRPQSSDNQQYFCAPAPLSPSARPRSPWGKLDPYDSSEDDKEYVGFATLPNQVHRKSVKK 122
```

Supplementary Figure 4. Alignment of N-terminal domains from SEPT9 and SEPT4 main isoforms. (**A**) SEPT9 isoforms, showing the basic (above) and acidic (below) domain division. (**B**) SEPT4 isoforms. The N-terminal domains of SEPT4 isoforms resemble, to some extent, those from SEPT9, despite being shorter.

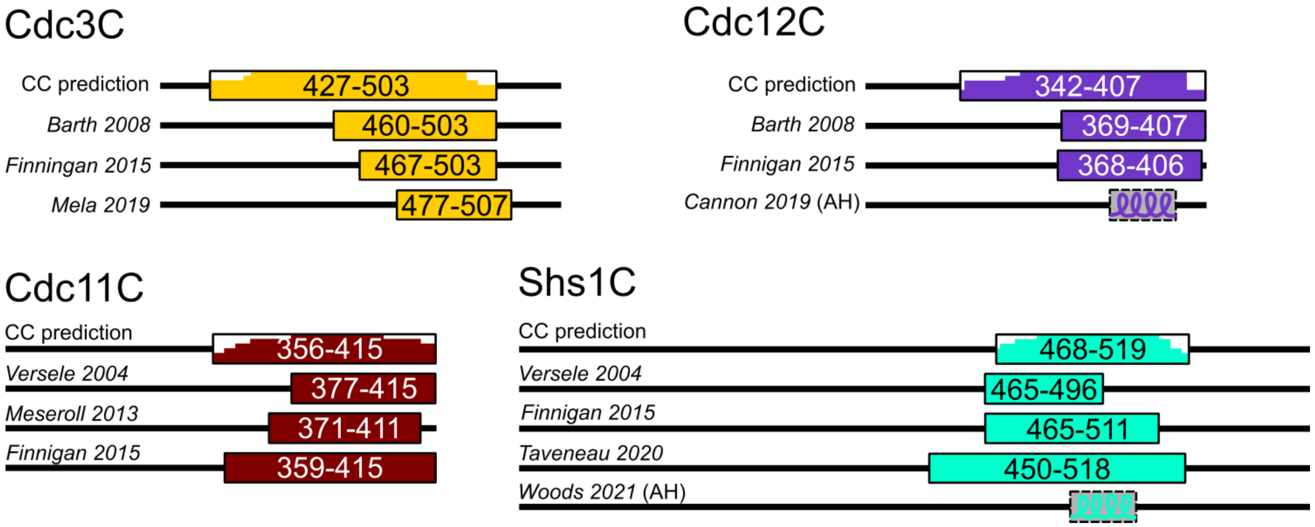

Supplementary Figure 5. Coiled coils (CCs) in baker's yeast (*Saccharomyces cerevisiae*) septins. Cdc10 have a short C-domain, no coiled-coil prediction and is not shown. CC region used by authors, together with 'consensus' coiled-coil predictions by COILS, Marcoil, DeepCoil and DeepCoil2. The degree of agreement in coiled-coil prediction among the five algorithms (sliding windows of 21 and 28 residues was used in COILS) is represented as the height of colored blocks within the line "CC prediction". Amphipathic helices (AHs) are likely found in Cdc12 and Shs1 and are represented in a grey dashed box.

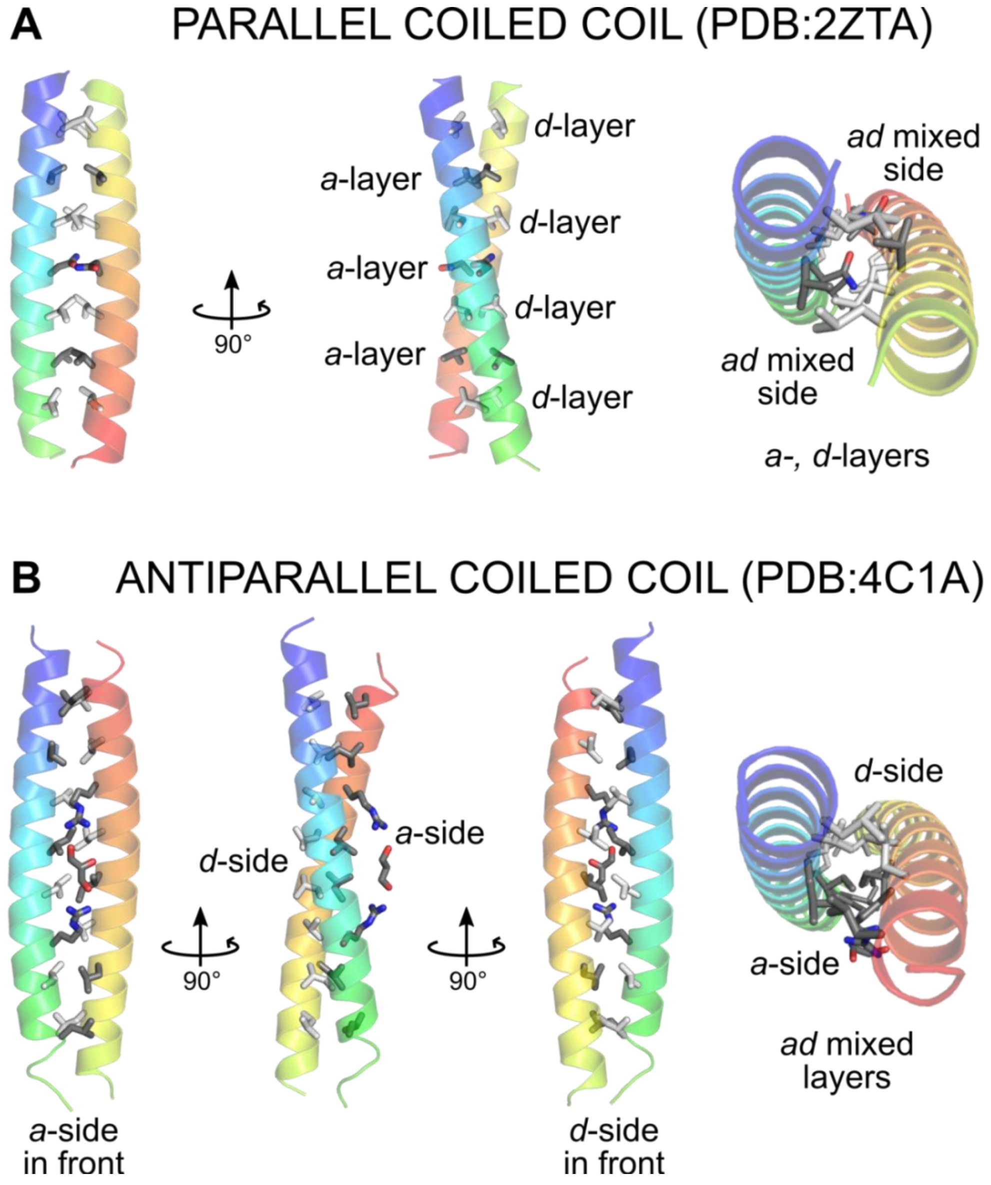

Supplementary Figure 6. Canonical dimeric coiled coils have distinct *a*,*d* layers/sides depending on the relative orientation of the helices. The *a* and *d* positions are represented in dark grey and white, respectively. (**A**) Parallel GCN4 coiled coil (PDB:2ZTA) has *a*-layers alternated with *d*-layers but *a*,*d*-mixed sides (e.g., *a* and *d* appear in both sides of the coiled coil). (**B**) Antiparallel ZfL2-1 coiled coil (PDB:4C1A) has, on the other hand, exclusive sides for *a* and *d* but *a*,*d*-mixed layers (a layer being composed of the core positions on the same height of the coiled coil).

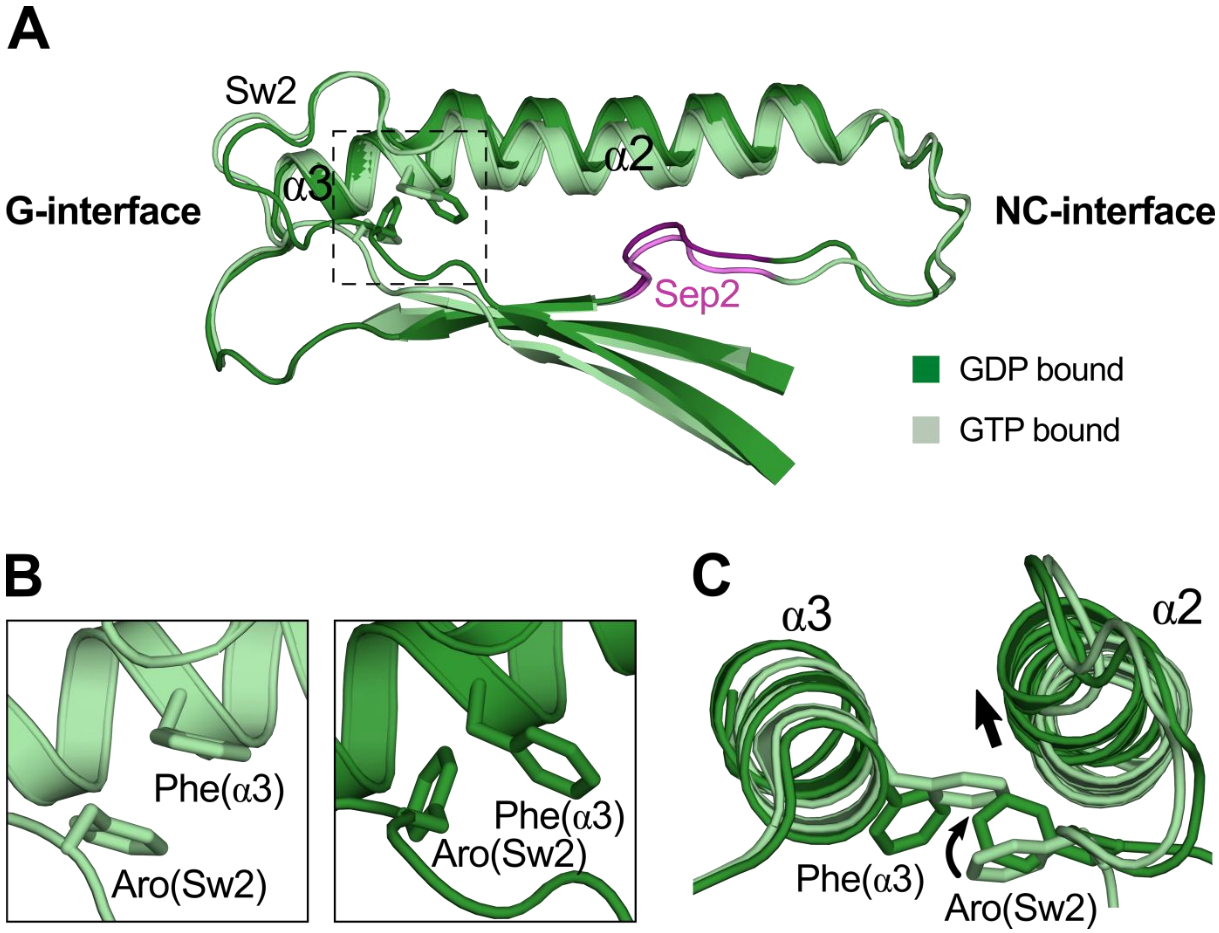

Supplementary Figure 7. Information transfer between adjacent interfaces and the likely role of Aro(Sw2) and Phe(α3). (**A**) Structural superposition of SEPT3 in the pre-hydrolysis (light green, SEPT3-GMPPNP, PDB:4Z51) and the post-hydrolysis (dark green, $SEPT3_{T282Y}$-GDP, PDB:6UQQ) states. Note that the α2 helix in the latter is slightly farther away from the β-sheet and Sep2 (highlighted in purple). Despite the model complexed with the GTP analogue (light green) does not form physiological G-interfaces within the crystal, the ordering of switch II indicates a *quasi*-physiological G-interface and represents at this moment the best possible model for the pre-hydrolysis state. (**B**) Snapshots of the aromatic cluster in the pre- and in the post-hydrolysis states. (**C**) Orientation seen from the G-interface. The rearrangement of Aro(Sw2) forces the α2 helix upwards.

2. Supplementary Tables

(this page was intentionally left blank)

Supplementary Table 1. Positions of relevant amino acid residues within each human septin. The residue is stated where there is no conservation. Uniprot entries are displayed below each sequence name. Asterisks (*) represent absent or unidentifiable positions.

Structural position	Human septins												
	**SEPT1** Q8WYJ6	**SEPT2** Q15019	**SEPT4** O43236	**SEPT5** Q99719	**SEPT7** Q16181	**SEPT6** Q14141	**SEPT8** Q92599-2	**SEPT10** Q9P0V9	**SEPT11** Q9NVA2	**SEPT14** Q6ZU15	**SEPT3** Q9UH03-2	**SEPT9** Q9UHD8-2	**SEPT12** Q8IYM1
Gly(HL)	12	19	126	26	32	24	26	48	23	34	43	262	31
Phe(HL)	13	20	127	27	33	25	27	49	24	35	Ile	Ile	Ile
Pro(α0)	17	24	131	31	37	29	31	53	28	39	Ile	Leu	Leu
Phe(α0)	29	36	143	43	49	41	43	65	40	51	60	279	48
Glu(P-loop)	38	45	152	52	58	50	52	74	49	60	Gln	Gln	Gln
Thr(P-loop/SEPT6)	Ser	Ser	Ser	Ser	Ser	51	53	75	50	61	Ser	Ser	Ser
Ser(P-loop)	44	51	158	58	64	56	58	80	55	66	75	294	63
Ala(Sw1/SEPT2)	64	71	178	78	Pro	*	*	*	*	*	Arg	Ser	Leu
His(Sw1/SEPT6)	*	*	*	*	*	74	76	98	73	84	*	*	*
Thr(Sw1)	71	78	185	85	90	Gly	Cys	Asn	Gly	Asn	102	321	89
Asp(Sw2)	94	101	208	108	113	Ser	103	Asn	100	Glu	125	344	112
GlyI(Sw2)	97	104	211	111	116	104	106	128	103	114	128	347	115
Aro(Sw2)	98^{Phe}	105^{Tyr}	212^{Phe}	112^{Phe}	117^{Phe}	105^{Phe}	107^{Phe}	129^{Phe}	104^{Phe}	115^{Tyr}	129^{Phe}	348^{Phe}	116^{Phe}
GlyII(Sw2)	99	106	213	113	118	106	108	130	105	116	130	349	117
Asx(Sw2)	103^{Asp}	110^{Asn}	217^{Asn}	117^{Asn}	122^{Asp}	110^{Asn}	112^{Asn}	134^{Asn}	109^{Asn}	120^{Asp}	134^{Asn}	353^{Asn}	121^{Asn}
Asn(Sw2)	Cys	Cys	218	118	123	Lys	Lys	Lys	Lys	Lys	135	354	122
Glu(α2)	126	133	240	140	145	133	135	157	132	143	157	376	144
Arg(PB2)	131	138	245	145	150	138	160	162	137	148	162	381	149
Phe(Tr1/SEPT2)	149	156	263	163	Ser	Thr	Thr	Thr	Thr	Thr	Thr	Thr	Thr
GlyI(Tr1)	150	157	264	164	169	159	161	183	158	169	182	401	169
His(Tr1)	Arg	158	265	165	170	160	162	184	159	170	183	402	170
GlyII(Tr1/SEPT2)	152	159	266	166	171	Ser	Ser	Ser	Ser	Ser	Ser	Ser	Cys
Leu(Tr1)	153	160	267	167	172	162	164	186	161	172	185	404	172
Ser(Tr1/SEPT6)	Pro	Pro	Pro	Pro	Pro	164	166	Thr	163	174	Pro	Pro	Pro
Leu(α3/SEPT6)	Val	Val	Val	Val	Ile	167	169	191	166	177	190	Ile	Ile
Phe(α3)	160	167	274	174	179	Thr	Thr	Thr	Thr	Thr	192	411	179
Asp(G4)	178	185	292	192	197	187	189	211	186	197	210	429	197
Glu(Tr2)	184	191	298	198	203	193	195	217	192	Asp	216	435	203
Glu(α4)	Gln	202	309	209	214	204	206	228	203	214	227	Asp	Asn
Pro(α4-α5′/SEPT3)	Gln	His	Gln	Gln	Glu	Gln	Gln	Gln	Gln	Gln	237	456	224
Gly(“G5”)	234	241	348	248	250	239	241	263	238	249	265	483	251
Arg(βb)	250	256	363	263	265	254	256	278	253	264	280	498	266
Glu(α6)	292	297	405	305	307	296	298	320	295	306	322	540	308
Arg(α6)	285	300	408	308	310	299	301	323	298	309	325	543	311

Supplementary Table 2. Positions of relevant amino acid residues within baker's yeast, fruit fly and *C. elegans* septins. The residue is stated where there is no conservation. Uniprot entries are displayed below each sequence name. Asterisks (*) represent absent or unidentifiable positions.

**Structural position**	***S. cerevisiae***					***D. melanogaster***					***C. elegans***	
	**Cdc3** P32457	**Cdc10** P25342	**Cdc11** P32458	**Shs1** Q07657	**Cdc12** P32468	**Sep1** P42207	**Sep4** Q7KUX3	**Sep2** P54359	**Sep5** Q7KLG8	**Pnut** P40797	**Unc-59** Q9U334	**Unc-61** Q25AR8
Gly(HL)	101	14	3	*	16	17	15	25	28	124	29	81
Phe(HL)	102	15	Ile	*	Ile	18	16	26	29	125	30	82
Pro(α0)	106	Thr	Ser	*	21	22	20	30	33	129	34	86
Phe(α0)	118	31	Ile	Ile	Gly	34	32	42	45	141	46	98
Glu(P-loop)	Pro	Gln	Gln	Pro	42	43	41	51	54	Ala	Arg	107
Thr(P-loop/SEPT6)	Asp	Ser	Ser	Ala	Ser	Ser	Ser	52	55	Ser	Ser	108
Ser(P-loop)	Thr	46	36	Thr	Thr	49	47	57	60	156	61	Thr
Ala(Sw1/SEPT2)	*	*	*	*	*	69	Val	*	*	Pro	*	*
His(Sw1/SEPT6)	*	*	*	*	*	*	*	75	78	*	*	131
Thr(Sw1)	Lys	74	Asp	Glu	75	76	74	Ser	Asn	183	89	135
Asp(Sw2)	204	97	89	Met	98	99	97	102	105	206	112	Glu
GlyI(Sw2)	207	100	92	138	101	102	100	105	108	209	115	161
Aro(Sw2)	208^{Phe}	101^{Phe}	93^{Phe}	Ile	102^{Phe}	103^{Phe}	101^{Phe}	106^{Tyr}	109^{Tyr}	210^{Phe}	116^{Phe}	162^{Phe}
GlyII(Sw2)	209	102	94	140	103	104	102	107	110	211	117	163
Asx(Sw2)	213^{Asn}	106^{Asp}	98^{Asp}	144^{Asp}	107^{Asn}	108^{Asp}	106^{Asn}	111^{Asn}	114^{Asn}	215^{Asp}	121^{Asn}	167^{Asp}
Asn(Sw2)	214	107	99	Asp	108	109	Cys	Lys	Lys	216	122	Lys
Glu(α2)	237	129	121	167	130	131	129	134	137	238	144	190
Arg(PB2)	242	134	126	172	135	136	134	139	142	243	149	195
Phe(Tr1/SEPT2)	Thr	Asn	Thr	Thr	Thr	154	Trp	Thr	Thr	Ser	Ser	Thr
GlyI(Tr1)	261	154	146	192	154	155	153	160	163	262	169	216
His(Tr1)	262	Lys	147	193	155	156	154	161	164	263	170	217
GlyII(Tr1/SEPT2)	Tyr	Glu	148	194	156	157	Ser	162	165	264	173	218
Leu(Tr1)	264	157	149	195	157	158	156	163	166	265	172	219
Ser(Tr1/SEPT6)	Pro	Arg	Glu	Glu	Pro	Pro	Gln	165	Ala	Pro	Pro	Ala
Leu(α3/SEPT6)	269	Val	Val	Val	Ile	Val	161	168	171	Ile	Ile	224
Phe(α3)	271	Ala	156	Leu	Thr	165	Leu	Cys	Cys	Cys	Leu	Thr
Asp(G4)	289	182	174	220	182	183	181	188	191	290	197	244
Glu(Tr2)	295	188	180	226	188	189	187	194	197	296	203	250
Glu(α4)	Gln	199	Asp	Asp	Val	200	Asp	205	208	307	Asp	261
Pro(α4-α5′/SEPT3)	Lys	209	Asn	Lys	Thr	210	Gln	Gln	Gln	Asp	Lys	Thr
Gly("G5")	344	236	230	Thr	247	239	237	240	243	345	251	296
Arg(βb)	360	251	247	288	263	254	252	255	258	360	266	312
Glu(α6)	402	293	289	330	305	295	294	297	300	402	308	354
Arg(α6)	405	296	292	333	308	298	297	300	303	405	311	357
